# β-catenin safeguards cell survival via a transcription-independent mechanism during the induction of primitive streak from hESCs

**DOI:** 10.1038/s41420-025-02559-w

**Published:** 2025-07-02

**Authors:** Peng Zhang, Xu-Xia Li, Hua-Jun Bai, Yongxu Zhao, Senle Rao, He Liang, Xiao-Ling Luo, Huang-Tian Yang

**Affiliations:** 1 Laboratory of Molecular Cardiology, CAS Key Laboratory of Tissue Microenvironment and Tumor, Shanghai Institute of Nutrition and Health, University of Chinese Academy of Sciences (CAS), CAS, Shanghai, China; 2 Translational Medical Center for Stem Cell Therapy & Institute for Heart Failure and Regenerative Medicine, Shanghai East Hospital, Tongji University School of Medicine and Shanghai Institute of Stem Cell Research and Clinical Translation, Shanghai, China; 3 Shandong Laboratory of Yantai Drug Discovery, Bohai Rim Advanced Research Institute for Drug Discovery, Yantai, Shandong China

**Keywords:** Embryonic stem cells, Cell signalling

## Abstract

The emergence of the primitive streak, representing an organizing center for gastrulation, marks the mesendodermal lineage specification from epiblast, in which the epiblast cells undergo highly organized collective behaviors to form mesendodermal cells properly. Cell death is observed at the peri-gastrulation stage, especially in the primitive streak region. However, the dynamic and regulatory mechanism of cell death in the primitive streak formation is unclear. Here, we observed that a quick inhibition of the fast elevated cell death is coinciding with an accumulation of β-catenin during the early stage of primitive streak induction from human embryonic stem cells (hESCs). Deficiency of β-catenin in hESCs does not affect their self-renewal but cause robust cell death after primitive streak induction, while neuroectodermal differentiation remains unchanged. Overexpression of full-length β-catenin in β-catenin-deficient hESCs restores the cell death restriction during induction of primitive streak. Mechanistically, the β-catenin-restricted cell death during primitive streak is transcription-independent. The accumulated β-catenin traps casein kinase-1 in β-catenin destruction complex following WNT activation via its ARM repeat domain, resulting in the inhibition of mTORC1 by stabilizing DEPTOR, subsequently attenuates mitochondrial translocation of p53 and enhances mitophagy to promote cell survival. Consistently, mTORC1 inhibition by rapamycin or RAD001 attenuates the cell death in β-catenin-deficient cells during induction of primitive streak. In addition, only the β-catenin retains activations of cell death restriction and transcriptional activity can promote hESCs to successfully differentiate into primitive streak and cardiomyocytes, suggesting that β-catenin-restricted cell death safeguards the fate transition during the primitive streak induction via offering a crucial window for the accumulation of β-catenin to induce lineage-specific genes. These findings provide new insights into the function and mechanisms by which β-catenin coordinates the cell death and early lineage commitment.

## Introduction

The forming of primitive streak (PS) is an initial event of gastrulation, which marks the onset of mesendodermal lineages [1]. The formation of PS requires orchestrated integration of morphogens gradients, intracellular signaling transduction, and downstream responses [2, 3]. Among various biological processes, regulation of cell death is one of the crucial issues [4]. Survival of epiblast cells during cell fate transition is the prerequisite to form its derivatives. Cell death has been observed at the early embryonic stage [5–7], with a transient increase at E6.0-E6.25 in mouse embryo (the time for the PS initiation) [5], and the dead cells are enriched near the midline of PS in chicken embryo [8]. Overreacted cell death results in embryonic lethality between E5.5–E6.5 in murine double minute 2 homolog (mdm2) deficient mice [9, 10], indicating that cell death should be tightly restricted in PS during early embryonic development. However, the correlation of cell death with the PS induction, and the underlying mechanism, particularly during the PS formation of human, are unclear.

WNT/β-catenin signaling is one of the critical pathways during PS formation [1, 11]. β-catenin is the non-redundant transducer in the WNT/β-catenin signaling to transfer signals to nucleus to initiate transcription of downstream genes [12]. The absence of β-catenin completely suppresses the expression of PS lineage genes in mouse embryonic stem cells (mESCs) [13]. Furthermore, detached cells from epiblast in the proamniotic cavity are observed in β-catenin deficient (β-cat^−/−^) mouse embryos between embryonic day (E) 6.5–7.0, followed by defect in PS formation [14]. This observation raises the possibility that β-catenin might involve in the regulation of cell death during early PS initiation. In addition, whether β-catenin participates in the regulation of cell death during PS formation of human, the influence of β-catenin-regulated cell death to PS formation, and the underlying mechanism are unknown.

β-catenin is strictly regulated by β-catenin destruction complex (consisting of the scaffolding protein AXIN, adenomatous polyposis coli (APC) protein, Ser/Thr kinases glycogen synthase kinase 3β (GSK-3β), casein kinase 1 (CK1), and E3-ubiquitin ligase β-TrCP). Upon the WNT activation, the β-catenin destruction complex becomes saturated by the phosphorylated β-catenin, and the excess β-catenin goes into nucleus to initiate expression of target genes [12]. The trapped components in the destruction complex also participate in multiple signaling pathways [15]. Thus, quenching the complex by β-catenin might cause the crosstalk between WNT/β-catenin signaling and other signaling pathways beyond the transcriptional activity of β-catenin, but it remains largely elusive in PS formation.

In this study, we aimed to determine the dynamic cell death during PS formation and elucidate its correlation with early PS commitment as well as the underlying mechanisms. Using the PS induction model from human ESCs (hESCs), we demonstrated the pivotal role of β-catenin in safeguarding PS formation via restricting cell death through β-catenin-CK1α-DEPTOR-mTORC1-p53 axis. The results also suggest that the death-restricted effect of β-catenin provides a critical time window for the accumulation of lineage-specific genes to accomplish the lineage transition. Overall, our findings uncover the novel function and regulatory mechanisms of β-catenin in the restriction of cell death and its significance to the process of early lineage commitment.

## Results

### Cell death during the early phase of PS induction from hESCs

hESCs were induced into cardiomyocytes (Fig. S1A). During this process, the cell fate transited from hESCs to functional PS lineages, cardiac mesoderm, cardiac progenitors, and eventually cardiomyocytes, which were characterized by the sequentially expressed lineage specific genes (Fig. S1B, C) and the highly purified cardiomyocytes (Fig. S1D) with organized sarcomere structure (Fig. S1E) and rhythmic beating (Video S1). These data are consistent with the previous report [16].

**Fig. 1:**
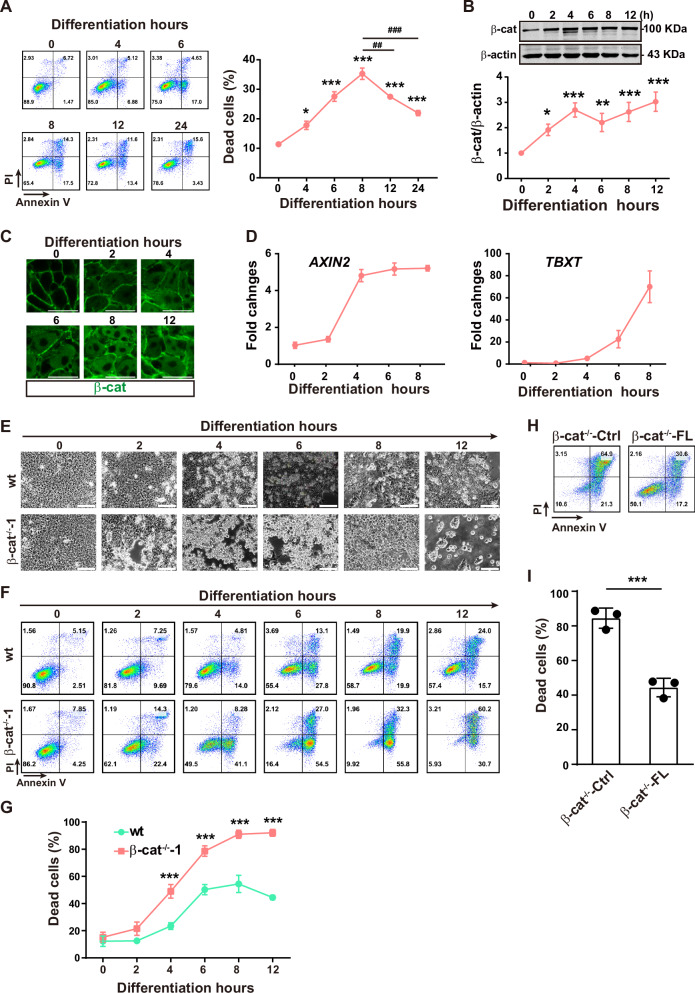
β-catenin is critical for cell survival during the early phase of PS induction from hESCs. **A** The representative plots (left panels) and the summarized data (right panels) of flow cytometry for detection of cell death in wild-type (wt) cells during PS induction. Data are represented as mean ± SEM; *n* = 3; *p < 0.05, ***p < 0.001 *vs*. undifferentiated cells (0 h group); ^##^*p* < 0.01, ^###^*p* < 0.001 *vs*. the indicated groups; one-way ANOVA followed by Tukey’s post hoc. **B** Western blot analysis of β-catenin during early PS induction. Data are represented as mean ± SEM; *n* = 5; *p < 0.05, **p < 0.01 *vs*. undifferentiated cells (0 h group); one-way ANOVA followed by Tukey’s post hoc. **C** Immunofluorescence staining of β-catenin during early PS induction. Scale bar, 5 μm. **D** qRT-PCR analysis of gene expression during PS induction. (**E**) Cell morphology during PS induction. Scale bar, 100 μm. **F** Representative flow cytometry plots of dead cell. (**G**) The summarized data of dead cells in (**F**). Data are represented as mean ± SEM; *n* = 3; *n.s*., non-significant; *p < 0.05, **p < 0.01, ***p < 0.001 by comparing with the wt cells at the same time point; two-way ANOVA followed by Sidak’s post hoc. **H** Flow cytometry analysis of β-cat^−/−^-Ctrl and β-cat^−/−^-FL cells by Annexin V and PI co-staining after 8-h of PS induction. **I** The summarized data from (H). Data are represented as mean ± SEM; *n* = 3; ***p < 0.001; Student’s t test.

Using this protocol, we examined the cell death during PS induction. The percentage of dead cells (defined by the sum of Annexin V/propidium iodide^+^ (AV^−^/PI^+^), AV^+^/PI^+^, and AV^+^/PI^−^ populations) was significantly elevated at 4 h, reached to the peak at 8 h, and dropped from 12 to 24 h (Fig. 1A). These data indicate that the transient increase of cell death during the early PS induction is restricted upon continuous differentiation.

### β-catenin is critical for the death restriction during the early phase of PS induction

During PS induction, the expression of β-catenin was upregulated at 2 h and reached to plateau from 4 h to at least 12 h (Fig. 1B, C), accompanying with the increased expression of *AXIN2* and *TBXT* (Fig. 1D). Because the restriction of cell death occurs following the robust accumulation of β-catenin, we speculated the involvement of β-catenin in it. To test this hypothesis, β-cat^−/−^ hESCs were generated by CRISPR/Cas9 from two parental hESC lines (β-cat^−/−^-1 and β-cat^−/−^-2 from H1, and β-cat^−/−^-3 from H7), which were confirmed by Sanger sequencing (Fig. S2A), western blot (Fig. S2B) and immunofluorescence staining (Fig. S2C). The clonal morphology, alkaline phosphatase (ALP) activity (Fig. S2D), and the expression of pluripotency markers OCT4 and SOX2 as well as the cell adhesion molecule E-cadherin (Fig. S2E) were comparable between the wt and β-cat^−/−^-1 cells, indicating that β-catenin deficiency does not affect the self-renewal and adhesion of hESCs.

Notably, robust cell death was observed in β-cat^−/−^-1 after PS induction (Fig. 1E–G). Similar phenotypes were observed in β-cat^−/−^-2 and β-cat^−/−^-3 cells (Fig. S3A, B) and various PS induction protocols (Fig. S3C). The causal relationship between the expression of β-catenin and cell death inhibition during early PS induction was further confirmed by lentivirus-mediated re-introduction of the full-length β-catenin (β-cat-FL) into the β-cat^−/−^-1 cells (β-cat^−/−^-FL). The expression of β-catenin was verified by immunofluorescence staining of β-catenin (Fig. S3D). The β-cat^−/−^-induced cell death was significantly inhibited in the β-cat^−/−^-FL cells (Fig. 1H, I). However, no differences in the cell morphology, cell death (Fig. S3E), and the expression of neuroectodermal genes (*SOX2*, *GBX2*, and *NEUROD1*) (Fig. S3F) were observed between the wt and β-cat^−/−^-1 cells during the neuroectodermal induction. These data demonstrate that β-catenin is necessary for the restriction of cell death during PS induction.

### β-catenin safeguards cell survival by inhibiting the mitochondrial pathway of p53 during PS induction

**Fig. 2:**
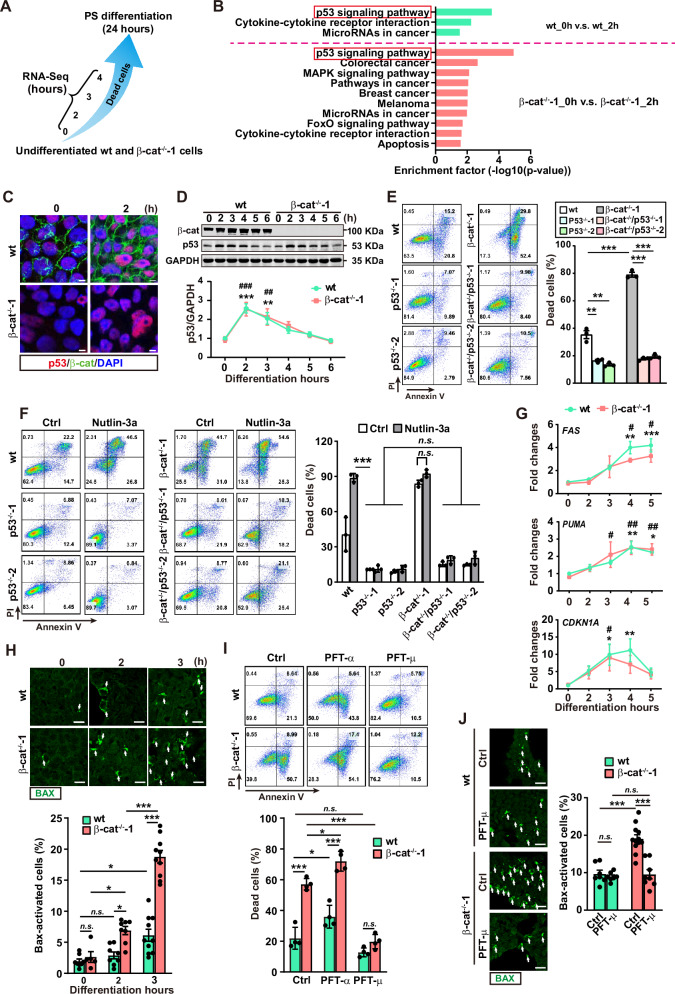
β-catenin safeguards the cell survival by inhibiting the proapoptotic mitochondrial apoptotic pathway of p53 during PS induction. **A** Schematic of selected time points for RNA-seq in wt and β-cat^−/−^-1 cells. **B** Significantly enriched signaling pathways. DEGs were defined by comparing the transcriptome at 2 h after differentiation with undifferentiated cells in both wt and β-cat^−/−^-1 cells. **C** Immunofluorescence staining of p53 (red) and β-catenin (green). Scale bar, 5 μm. **D** The expression of β-catenin and p53 during PS induction examined by western blot. GAPDH is used as the internal control. Data are represented as mean ± SEM; *n* = 5; **p < 0.01, ***p < 0.001 *vs*. 0 h in the wt cells; ^##^*p* < 0.01, ^###^*p* < 0.001 *vs*. 0 h in the β-cat^−/−^-1 cells; one-way ANOVA followed by Tukey’s post hoc. **E** Flow cytometry analysis of dead cells at 8 h after PS induction in the p53 knockout (p53^−/−^) cells. Data are represented as mean ± SEM; *n* = 3; **p < 0.01, ***p < 0.001*vs*. indicated groups; two-way ANOVA followed by Sidak’s post hoc. **F** The representative plots (left panels) and the summarized data (right panels) of flow cytometry analysis of dead cells detected at 8 h after PS induction with or without the nutlin-3a treatment. Data are represented as mean ± SEM; *n* = 3; *n.s*., non-significant, ***p < 0.001; two-way ANOVA followed by Sidak’s post hoc. **G** qRT-PCR analysis of the expression of *FAS*, *PUMA* and *CDKN1A* during PS induction. Data are represented as mean ± SEM; *n* = 3; *p < 0.05, **p < 0.01, ***p < 0.001 *vs*. 0 h in the wt cells; ^#^*p* < 0.05, ^##^*p* < 0.01 *vs*. 0 h in the β-cat^−/−^-1 cells; one-way ANOVA followed by Tukey’s post hoc. (**H** Immunofluorescence analysis of BAX activation (white arrows) in wt and β-cat^−/−^-1 cells during early induction of PS. Scale bar, 20 μm. Data are represented as mean ± SEM; *n* = 5-10; *n.s*., non-significant, *p < 0.05, ***p < 0.001; two-way ANOVA followed by Sidak’s post hoc. **I** Flow cytometry analysis of dead cells in the wt and β-cat^−/−^-1 cells treated with PFT-α or PFT-μ. The cells were analyzed at 4 h after PS induction. Data are represented as mean ± SEM; *n* = 4; *n.s*., non-significant, *p < 0.001, ***p < 0.001; two-way ANOVA followed by Sidak’s post hoc. **J** Immunofluorescence analysis of BAX activation (white arrows) in the wt and β-cat^−/−^-1 cells treated with PFT-μ during early induction of PS. Scale bar, 20 μm. Data are represented as mean ± SEM; *n* = 7-12; *n.s*., non-significant, ***p < 0.001; two-way ANOVA followed by Sidak’s post hoc.

Next, the apoptosis-related protein array was conducted to explored the molecular mechanism underlying the β-catenin-dependent death restriction. No differentially expressed proteins between the wt and β-cat^−/−^-1 cells at 2 h of PS induction were identified (Fig. S4A, B). Then we performed RNA-sequencing (RNA-seq) in the wt and β-cat^−/−^-1 cells at 0, 2, 3, and 4 h of PS induction (Fig. 2A). Kyoto Encyclopedia of Genes and Genomes (KEGG) analysis of the differentially expressed genes (DEGs) revealed that the p53 signaling pathway was significantly enriched in the upregulated DEGs by comparing the transcriptome of the cells at each differentiation time point with the undifferentiated ones in both wt and β-cat^−/−^-1 cells (Figs. 2B and S4C). Consistently, p53 significantly accumulated at 2 h of PS induction in immunofluorescence staining and western blot (Fig. 2C, D) and gradually returned to baseline at 6 h of PS induction (Fig. 2D).

To determine the role of p53 in the β-catenin-dependent death restriction, p53 deficient hESCs from H1-wt hESCs (p53^−/−^-1, p53^−/−^-2 from H1-wt) and β-cat^−/−^-1 hESCs (β-cat^−/−^/p53^−/−^-1 and β-cat^−/−^/p53^−/−^-2 from β-cat^−/−^-1 hESCs) were generated by CRISPR/Cas9. The mutations were validated by Sanger sequencing (Fig. S4D), and the absence of p53 was confirmed by western blot (Fig. S4E). All p53 deficient hESCs showed similar morphology, ALP activity, and expression of pluripotency genes with the parental cells (Fig. S4F, G), indicating that self-renewal of hESCs is not affected. However, the percentages of dead cells were significantly reduced in all p53^−/−^ cells during PS induction (Fig. 2E). Similar phenotypes were observed even under the treatment of nutlin-3a (an activator of p53 [17]) (Fig. 2F). These results indicate that β-catenin suppresses the cell death during PS induction by inhibiting p53.

p53 promotes cell death via the transcription-dependent (activating the expression of pro-death genes) and transcription-independent mechanism (promoting the mitochondrial apoptosis) [18, 19]. The expression of the p53 downstream genes (*FAS*, *PUMA* and *CDKN1A*) were significantly upregulated during PS initiation but were comparable between the wt and β-cat^−/−^ -1 cells (Fig. 2G), while the percentages of cells with activated BAX, a classic mitochondrial apoptosis marker and a direct target of p53 in promoting mitochondrial apoptosis [20], were markedly higher at 2 and 3 h during PS induction in the β-cat^−/−^-1 cells comparing with the wt cells (Fig. 2H). Moreover, treating cells with pifithrin-μ (PFT-μ, an inhibitor of p53-depedent mitochondria pathway [21]), but not pifithrin-α (PFT-α, an inhibitor of p53 transcriptional activity[22]), blocked the cell death in the β-cat^−/−^-1 cells (Fig. 2I). Concomitantly, the enhanced percentage of BAX-activated β-cat^−/−^-1 cells was reversed to the wt cell level after treated with PFT-μ, but no alteration was detected in the wt cells treated with PFT-μ at 3 h of PS induction (Fig. 2J). These data suggest that β-catenin inhibits the p53-depedent mitochondria pathway to safeguard the survival of cells during PS induction.

### β-catenin transcription-independently inhibits cell death

**Fig. 3:**
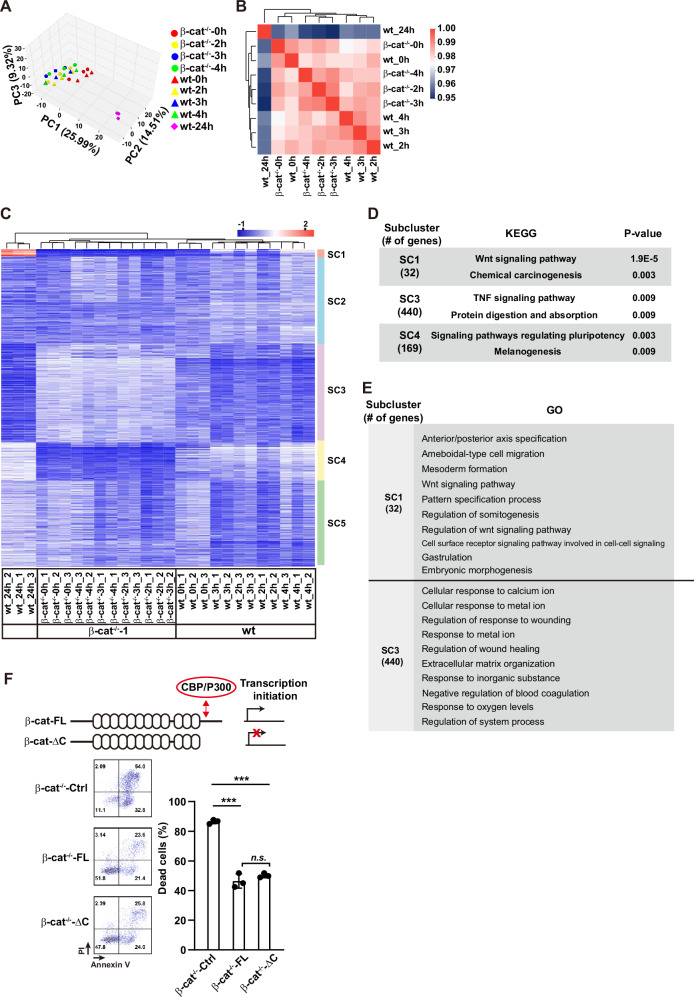
β-catenin inhibits apoptosis during PS induction in a transcription-independent way. **A** PCA plots of RNA-seq. **B** Spearman correlation of RNA-seq datasets. **C** Heatmap of DEGs by comparing the expression of genes in wt and β-cat^−/−^-1 cells at 0, 2, 3, 4 h after PS induction. The sub-clusters (SC) were indicated on the right. **D** Enriched pathways revealed by KEGG analysis of genes in each SC in (**C**). **E** GO analysis of genes in each SC in (**D**). **F** Flow cytometry analysis of cell death after 8-h of PS induction in β-cat^−/−^-Ctrl, β-cat^−/−^-FL, and β-cat^−/−^-ΔC cells. Data are represented as mean ± SEM; *n* = 3; *n.s*., non-significant; ***p < 0.001; one-way ANOVA followed by Tukey’s post hoc.

In the classic view, β-catenin promotes PS formation via promoting the expression of lineage-specific genes [23]. We thus explored the downstream genes of β-catenin modulating p53 activity. PCA and correlation assay indicated the high similarity of transcriptomes of cells at 2, 3, and 4 h after PS induction as well as the undifferentiated cells between the wt and β-cat^−/−^-1 cells, except the wt cells at 24 h of differentiation were significantly separated from others (Fig. 3A, B).

To sort out the key gene(s) regulating the p53 signaling, we did the enrichment analysis of biological process enrichment of gene ontology (GO-BP) and KEGG pathways of DEGs between the wt and β-cat^−/−^ cells at 2, 3 and 4 h of differentiation, respectively. No enriched GO-BP term or KEGG pathways were found for the DEGs between the wt and β-cat^−/−^ cells at differentiation 2 h (data not shown) nor related to “p53” or “apoptosis” for the DEGs at differentiation 3 or 4 h (Figs. S5, S6, and Table S1).

Next, two strategies were used to explore the possible target genes. In strategy 1, the DEGs were identified by comparing the transcriptome of the cells at tested time points with the undifferentiated cells. A total of 2998 genes, with fold changes >2 and p-adjust < 0.05, were identified and clustered into five subclasses (SCs) (Fig. S7A). The expression profile of DEGs in SC4 differed between wt and β-cat^−/−^-1 cells, while mild changes were observed in other SCs (Fig. S7A). The p53 signaling pathway was enriched in SC2 (Fig. S7B), however, the expression of related genes was comparable between the wt and β-cat^−/−^-1 cells (Fig. S7C). Moreover, no pathways related with cell death in other SCs were enriched (Fig. S7B).

Because no specific genes responsible for the β-catenin-dependent restriction of cell death were identified using this strategy, we examined the DEGs by comparing the transcriptome between wt and β-cat^−/−^-1 cells at the same differentiation time. 1411 DEGs were identified. These DEGs were grouped into 5 SCs (Fig. 3C). WNT signaling is the most enriched pathway in SC1 (Fig. 3D), while the related genes were enriched in the regulation of early lineage specification as revealed by GO analysis (Fig. 3E). No item related to “cell death” or “apoptosis” were identified in the top100 enriched results in all of 5 SCs (Fig. 3E and Table S2). Taken together, no gene(s) showed a strong correlation with the β-catenin-dependent restriction of cell death. We, thus, speculate that the transcriptional activity of β-catenin may not be responsible for the cell death restriction during the early phase of PS induction.

To test this hypothesis, β-cat^−/−^-1 cells were infected with lentivirus containing β-cat-FL and β-catenin without C-terminal domain (β-cat-ΔC, which loss its transcriptional activity [13]), respectively. Their expressions were confirmed by western blot (Fig. S7D) and immunofluorescence staining (Fig. S7E). The expression of downstream genes *T* and *AXIN2* were rescued by β-cat-FL but not by the control vector or β-cat-ΔC (Fig. S7F), which confirmed the transcriptional impairment in the β-cat^−/−^-1 cells containing β-cat-ΔC (β-cat^−/−^-ΔC cells). However, the expression of β-cat-FL and β-cat-ΔC both attenuated the cell death after 8 h of PS induction (Fig. 3F). These results strongly suggest that β-catenin inhibits cell death in a transcription-independent way during the early phase of PS induction.

### β-catenin suppresses cell death via inhibition of mTORC1 during the early phase of PS induction

**Fig. 4:**
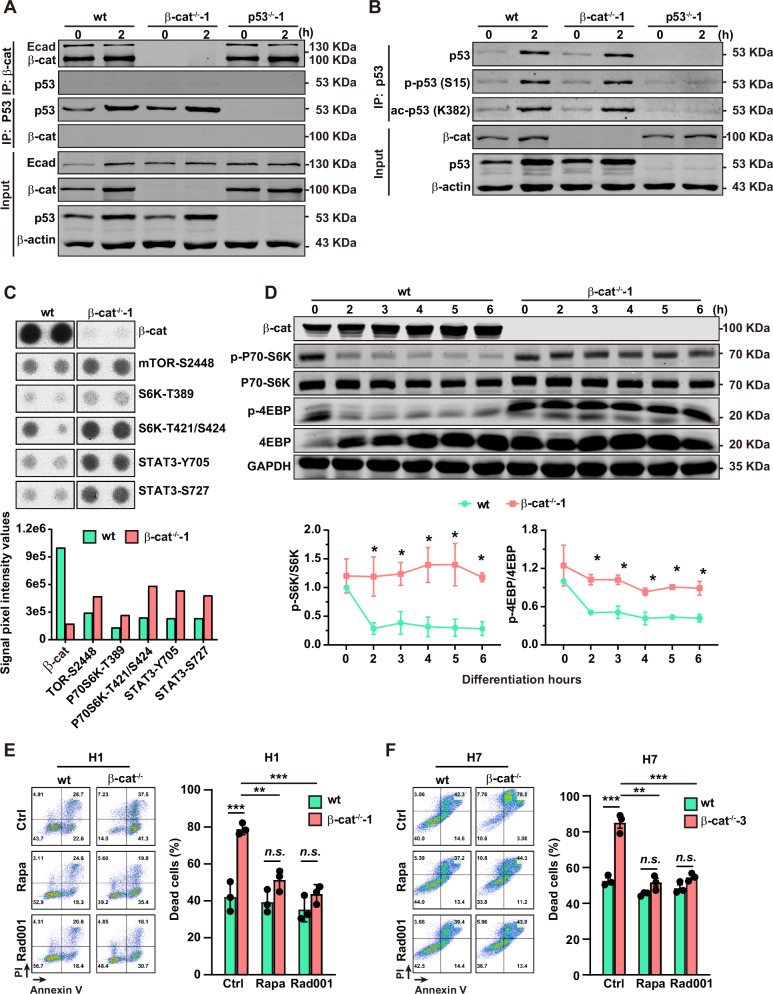
β-catenin suppresses cell death during early phase of PS specification via inhibition of mTORC1. **A** Co-IP for interaction analysis of β-catenin and p53 in wt, β-cat^−/−^-1 cells at 0 and 2 h after PS induction. The 0-h group, the undifferentiated hESCs; p53^−/−^-1 cells were used as the control. Ecad, E-cadherin, a marker for successful co-IP of β-catenin. β-actin is used as the internal control. **B** Post-translational modifications of the phosphorylation at Ser15 and acetylation at Lys382 of p53 was detected by western blot. **C** Proteins with changed phosphorylation modifications reveled by protein kinases array analysis of wt and β-cat^−/−^-1 cells at 2 h after PS induction. **D** The representative images (upper panel) and the summarized data (lower panels) for the protein levels of phosphorylated P70-S6K and 4EBP during PS induction in wt and β-cat^−/−^-1 cells. Data are represented as mean ± SEM; *n* = 3; *p < 0.05, wt *vs*. β-cat^−/−^-1; one-way ANOVA followed by Tukey’s post hoc. **E** Representative (left panels) and summarized data (right panels) of flow cytometry analysis of cell death with addition of rapamycin or RAD001 in H1 cells. The small molecules were used at 10 nmol/L. Data are represented as mean ± SEM; *n* = 3; *n.s*. non-significant; ***p < 0.001; two-way ANOVA followed by Sidak’s post hoc. **F** Representative (left panels) and summarized data (right panels) flow cytometry analysis of cell death with addition of rapamycin or RAD001 in H7 cells. The small molecules are used at 10 nmol/L. Data are represented as mean ± SEM; *n* = 3; *n.s*., non-significant; ***p < 0.001; two-way ANOVA followed by Sidak’s post hoc.

We next test whether β-catenin inhibits p53 by direct protein-protein interaction. No interaction of p53 and β-catenin was detected by analyses with either co-immunoprecipitation (Co-IP) following liquid chromatography mass spectrometry (LC-MS) (Table S3) or western blot (Fig. 4A). The two critical PTM sites (Ser-15 (S15) phosphorylation and Lys-382 (K382) acetylation of p53 [24]) were also comparable between the wt and β-cat^−/−^-1 cells (Fig. 4B) at 2 h of PS induction. These data support the indirect intervention of p53 by β-catenin.

Next, the human phospho-kinase array was preformed to explore the dysregulated pathways related to β-catenin deficiency during PS induction (Fig. S6). The phosphorylation level of most tested proteins was comparable between the wt and β-cat^−/−^-1 cells after 2 h of induction, including AMPK, p38, ERK, and AKT (Fig. S8). Intriguingly, the phosphorylation level of mTOR-Ser2448, P70S6K-Thr389, P70S6K-Thr421/Ser424, STAT3-Ser727, and STAT3-Tyr705 were significantly higher in the β-cat^−/−^-1 cells (Figs. 4C and S6). The phosphorylation level of these sites were reported to be positively correlated with mTORC1 activity [25–28], which suggests that mTORC1 activity is abnormally higher in β-catenin-deficient cells during PS induction, whereas AMPK, p38, ERK, and AKT signaling pathways are less responsible for the abnormal mTORC1 activity. Consistent with the array data, the phosphorylation of P70-S6K and 4EBP, the downstream targets of mTORC1 [29], were rapidly downregulated after PS induction in wt cells, while they stably maintained at a high level in β-cat^−/−^-1 cells (Fig. 4D). In addition, the β-catenin-deficiency-related cell death in the β-cat^−/−^-1 cells during PS induction was significantly inhibited by mTORC1 inhibitors rapamycin and RAD001 in both β-cat^−/−^-1 and β-cat^−/−^-3 cells (Fig. 4E, F). Taken together, these data indicate β-catenin-restricted cell death during the early phase of PS differentiation via inhibiting mTORC1 activity.

### β-catenin attenuates mitochondrial translocation of p53 and promotes mitophagy via inhibition of mTORC1

**Fig. 5:**
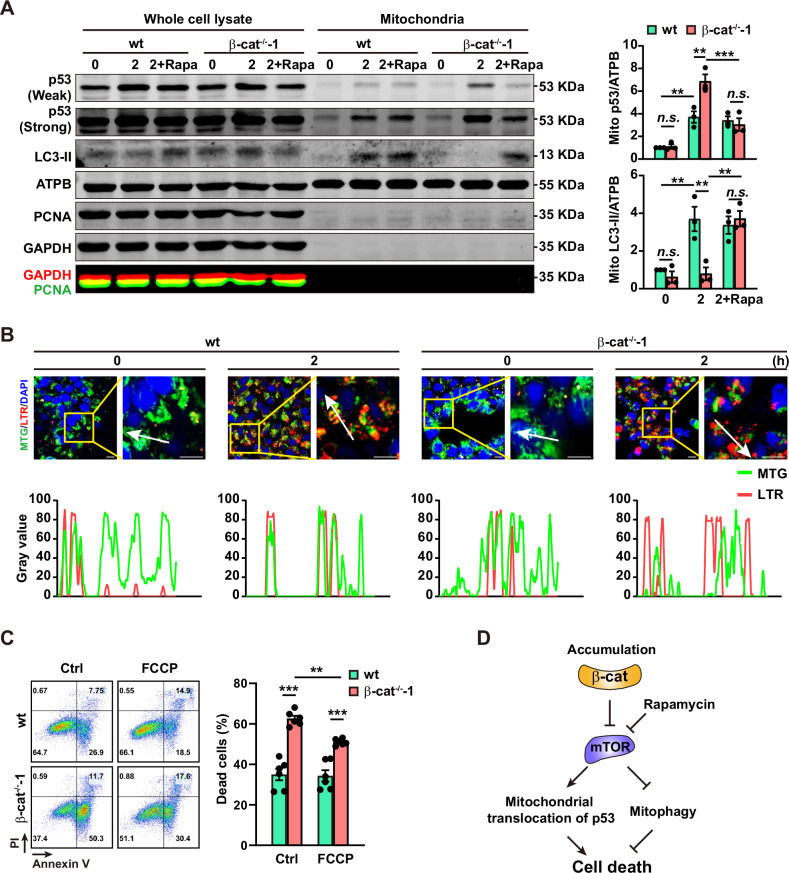
β-catenin attenuates mitochondrial translocation of p53 and promotes mitophagy via inhibition of mTORC1 during PS induction. **A** Representative (left panel) and summarized data (right panels) of western blot analysis of p53 and LC3-II in mitochondria after 2 h of PS induction. ATPB, the mitochondria marker. PCNA, the nuclear marker. GAPDH, the cytoplasm marker. 0 and 2, the PS induction time (h); Rapa, rapamycin treated at 10 nmol/L; 2+Rapa, the cells treated with rapamycin for 2 h since the PS induction. Data are represented as mean ± SEM; *n* = 3; *n.s*., non-significant; **p < 0.01, ***p < 0.001; two-way ANOVA followed by Sidak’s post hoc. **B** MitoTracker Green (MTG) and LysoTracker Red (LTR) staining in wt and β-cat^−/−^-1 cells at 0 (undifferentiated stage) and 2 h after PS induction. **C** Representative (left panels) and summarized data (right panels) of Flow cytometry analysis of cell death in wt and β-cat^−/−^-1 cells with or without FCCP (1 μmol/L) treatment by Annexin V and PI co-staining. The cells are harvested after 8-h of PS induction. Data are represented as mean ± SEM; *n* = 3; **p < 0.01, ***p < 0.001; two-way ANOVA followed by Sidak’s post hoc. **D** The schematic of how mTORC1 inhibition promotes cell survival during PS induction.

The translocation of p53 to mitochondria triggers the initiation of apoptosis [19]. In the high-purity mitochondria, marked by the absence of the nuclear marker proliferating cell nuclear antigen (PCNA) or cytoplasm marker GAPDH, the mitochondrial p53 was significantly enhanced in the β-cat^−/−^-1 cells comparing with the wt cells at 2 h of PS induction, but it was attenuated by rapamycin (Fig. 5A) or PFT-μ treatment (Fig. S9).

Mitophagy is reported to protect the cell by elimination of damaged mitochondria [30] and it is negatively regulated by mTORC1 [31]. At 2 h of PS induction, the elevation of mitochondrial LC3-II was inhibited in the β-cat^−/−^-1 cells (Fig. 5A), suggesting that the increased mitophagy is crippled. Treating β-cat^−/−^-1 cells with rapamycin restored the mitochondrial LC3-II (Fig. 5A). In addition, the colocalization of MitoTracker green and LysoTracker red was largely disrupted in β-cat^−/−^-1 cells at 2 h after PS induction (Fig. 5B). Moreover, FCCP, a commonly used mitophagy inducer [32], promoted cell survival in β-cat^−/−^-1 cells (Fig. 5C). Taken together, the accumulated β-catenin suppresses mTORC1, thereby inhibiting p53 mitochondrial translocation, promoting mitophagy, and ensuring cell survival during the early phase of PS induction (Fig. 5D).

### The armadillo (ARM) repeat domain of β-catenin is responsible for the inhibition of mTORC1 via stabilizing DEPTOR through CK1α blockage

**Fig. 6:**
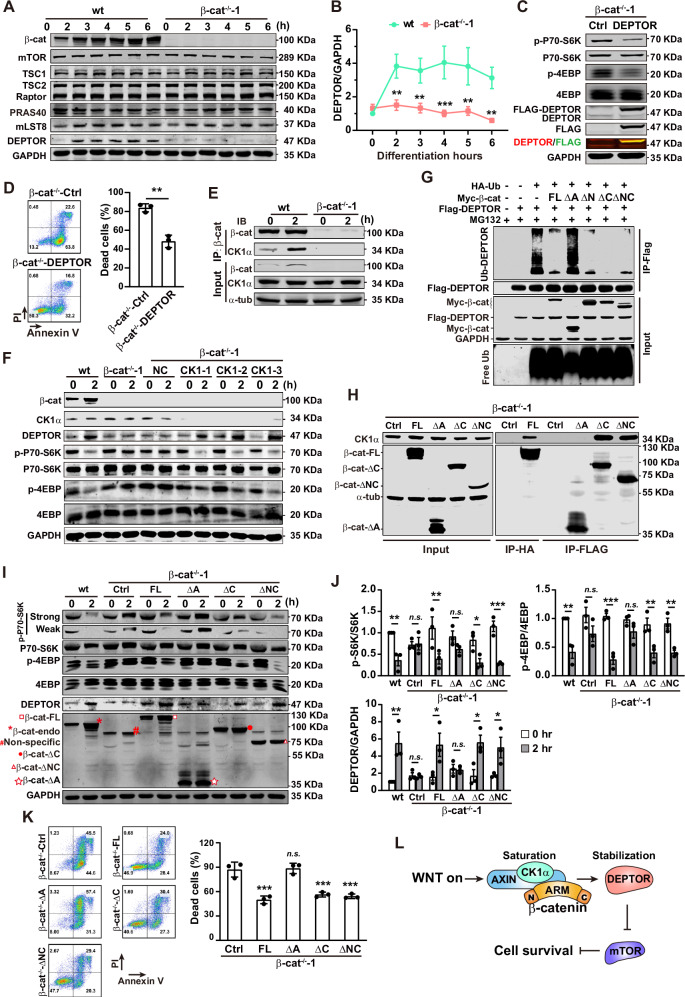
The armadillo repeat domain of β-catenin is responsible for mTORC1 inhibition by stabilizing of DEPTOR through CK1α blockage during PS induction. **A** Western blot analysis of the expression of mTOR, TSC1, TSC2, Raptor, PRAS40, mLST8, and DEPTOR during early PS induction in wt and β-cat^−/−^-1 cells. GAPDH is used as the internal control. **B** Normalized DEPTOR expression during early PS induction. Data are represented as mean ± SEM; *n* = 3; **p < 0.01, ***p < 0.001 *vs*. the wt group at the same time points; two-way ANOVA followed by Sidak’s post hoc. **C** Western blot analysis of mTORC1 activity in β-cat^−/−^-1 cells infected with the control virus (β-cat^−/−^-Ctrl) or the virus expressing DEPTOR (β-cat^−/−^-DEPTOR). **D** Flow cytometry analysis of β-cat^−/−^-Ctrl and β-cat^−/−^-DEPTOR cells at 8 h of PS induction. The representative plots (left panels) and summarized data (right panels). Data are represented as mean ± SEM; *n* = 3; **p < 0.01; Student’s *t* test. **E** Co-IP to detect the interaction of β-catenin and CK1α. The α-tubulin is used as the internal control. **F** Western blot analysis of the expression of DEPTOR and mTORC1 activity in the CK1α downregulated cells. 3 different siRNAs (CK1-1, CK1-2, CK1-3) targeting CK1α are used. **G** Polyubiquitinated DEPTOR detection in HEK293T cells co-transfected of DEPTOR with different versions of β-catenin expression plasmids. The MG132 (50 μmol/L) is used to inhibit the degradation of DEPTOR. **H** Co-IP of CK1α with β-cat-FL, β-cat-ΔA, β-cat-ΔC, and β-cat-ΔNC in β-cat^−/−^-1 cells at 2 h of PS induction. **I** Detection of mTORC1 activity in wt, β-cat^−/−^-Ctrl, β-cat^−/−^-FL, β-cat^−/−^-ΔA, β-cat^−/−^-ΔC, and β-cat^−/−^-ΔNC cells. The undifferentiated cells and the cells at 2 h of PS induction are harvested and analyzed. For detect the different form of β-catenin, anti-β-catenin and anti-FLAG antibodies were co-incubated with the membrane. Strong: the fluorescent data collected by lI-COR Odyssey Imagers are proceed to enhance the visual effect of the weak bands; weak: the fluorescent data collected by lI-COR Odyssey Imagers are proceed to reduce the visual effect of the strong bands. The processes do not affect the quantification of each band. Red square, β-cat-FL; Red asterisk, endogenous β-catenin; octothorpe: non-specific band of anti-β-catenin antibody (BD); red solid circle, β-cat-ΔC; red outlined triangle, β-cat-ΔNC; red outlined pentagram, β-cat^−/−^-ΔA. **J** The normalized data of (**I**). Data are represented as mean ± SEM; *n* = 3; *n.s*., non-significant; **p < 0.01, ***p < 0.001; two-way ANOVA followed by Sidak’s post hoc. **K** Representative plots (left panels) and summarized data (right panels) of flow cytometry analysis for cell death detection in β-cat^−/−^-Ctrl, β-cat^−/−^-FL, β-cat^−/−^-ΔA, β-cat^−/−^-ΔC, and β-cat^−/−^-ΔNC cells after 8 h of PS induction. Data are represented as mean ± SEM; *n* = 3; *n.s*., non-significant, ***p < 0.001 *vs*. the Ctrl group; one-way ANOVA followed by Tukey’s post hoc. **L** Snapshot of the β-catenin-mediated inhibition in mTOR during PS induction.

To elucidate the mechanism underlying β-catenin-mediated mTORC1 inhibition, we examined the expression of mTORC1 components, including the regulatory-associated protein of mTOR (Raptor), mammalian lethal with Sec13 protein 8 (mLST8), proline-rich AKT substrate 40 kDa (PRAS40), DEPTOR, and the key mTORC1 regulatory protein TSC1/2. The level of these proteins maintained unchanged during PS induction in both wt and β-cat^−/−^-1 cells, except DEPTOR, which was significantly upregulated and reached the maximal level from 2 h in the wt cells, while maintained at a low level in β-cat^−/−^-1 cells (Figs. 6A, B and S10A). Co-expression of β-catenin with each mTORC1 component in HRK293T cells promoted the expression of DEPTOR, but not PRAS40, mLST8, and Raptor (Fig. S10B). The profile of DEPTOR expression inversely matched with the changes of mTORC1 activity (Fig. 4D), which is consistent with the previous finding that DEPTOR is the inhibitory element in mTORC1 [33]. Indeed, overexpression of DEPTOR in β-cat^−/−^-1 cells resulting in the inhibition of mTORC1 activity (Fig. 6C) and restriction of cell death (Fig. 6D). These data demonstrate that β-catenin inhibits mTORC1 activity by elevating the level of DEPTOR during early PS induction.

Next, we investigated how β-catenin elevates the level of DEPTOR. The transcription of DEPTOR was comparable between the wt and β-cat^−/−^-1 cells as revealed by RNA-seq analysis (Fig. S10C). The rapid elevation of DEPTOR suggests that accumulated β-catenin may protect DEPTOR from degradation. DEPTOR is degraded by βTrCP after polyubiquitination following the priming phosphorylation by CK1α [34, 35] and CK1α is a key component in the β-catenin degradation complex, which is saturated by phospho-β-catenin after WNT activation [12]. We then tested the hypothesis that β-catenin stabilizes DEPTOR by trapping CK1α in the β-catenin degradation complex after WNT activation and it is supported by the evidences: (1) the enhanced interaction between β-catenin and CK1α at 2 h of PS differentiation in the wt cells (Fig. 6E); and (2) the elevation of DEPTOR and inhibition of mTORC1 in β-cat^−/−^-1 cells transfected with three distinct siRNAs targeting CK1α (Fig. 6F).

β-catenin contains a central ARM domain, an N-terminal domain, and a C-terminal domain, and these domains were characterized with different functions [36]. Various truncated β-catenin were used to identify the key domain responsible for the inhibition of mTORC1. The protein level of DEPTOR was significantly enhanced with the co-transfection of β-cat-FL, β-cat-ΔC, N-terminal-deleted (β-cat-ΔN), and both N- and C-terminal-deleted (β-cat-ΔNC) β-catenin but not β-catenin with ARM domain-deleted (β-cat-ΔA) (Fig. S10D). Moreover, CK1α was co-precipitated with β-cat-FL, β-cat-ΔC, β-cat-ΔN, and β-cat-ΔNC but not with β-cat-ΔA in HEK293T cells (Fig. S10E). Consistently, polyubiquitinated DEPTOR was reduced in the cells co-expressing β-catenin variants containing ARM domain (Fig. 6G). These data demonstrate that the ARM domain of β-catenin is responsible for the CK1α blockage, which promotes the stabilization of DEPTOR, resulting in the inhibition of mTORC1.

We further verified the above mechanism by overexpressing different forms of β-catenin in the β-cat^−/−^-1 cells. The β-cat^−/−^-Ctrl, β-cat^−/−^-FL, β-cat^−/−^-ΔA (β-cat^−/−^-1 with β-cat-ΔA overexpression), β-cat^−/−^-ΔC, and β-cat^−/−^-ΔNC (β-cat^−/−^-1 with β-cat-ΔNC overexpression) cells were generated and displayed similar clonal morphology as the control ones (Fig. S10F). However, the β-cat^−/−^-ΔN (β-cat^−/−^-1 with β-cat-ΔN overexpression) cells underwent spontaneously differentiation (Fig. S10F), which is likely due to the constant activation of the β-catenin without its N-terminal domain [37]. The expression of various forms of β-catenin were further confirmed by immunofluorescence staining (Fig. S10G). CK1α was co-precipitated with β-cat-FL, β-cat-ΔC, and β-cat-ΔNC but not β-cat-ΔA in hESCs (Fig. 6H). DEPTOR was accumulated at 2 h after PS induction in the wt, β-cat^−/−^-FL, β-cat^−/−^-ΔC, and β-cat^−/−^-ΔNC cells, accompanying with the mTORC1 inhibition and cell death restriction, but not in the β-cat^−/−^-Ctrl and β-cat^−/−^-ΔA cells (Fig. 6I–K). Together, these data demonstrate that during PS formation, the accumulated β-catenin stabilizes DEPTOR via trapping CK1α in β-catenin degradation complex, and subsequently inhibits mTORC1 to safeguard cell survival (Fig. 6L).

### Proper PS induction requires coordination of β-catenin-mediated cell survival and transcription activity

**Fig. 7:**
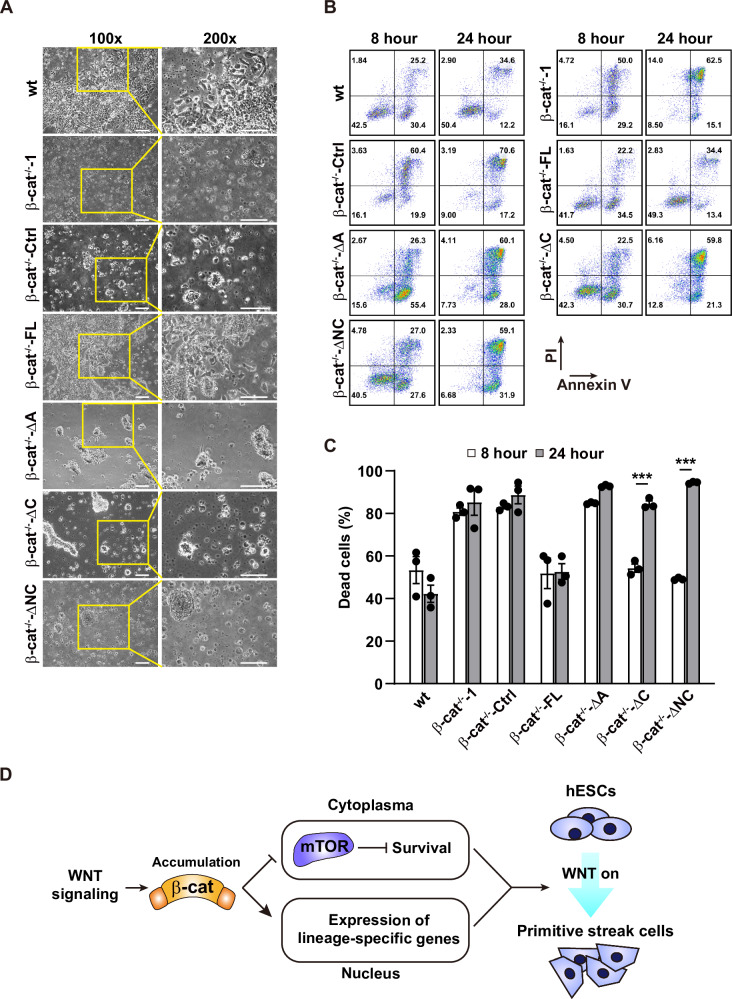
Proper PS induction requires coordination of the transcription-independent cell death inhibition and the transcription-dependent activity of β-catenin. **A** Cell morphology of wt, β-cat^−/−^-Ctrl, β-cat^−/−^-FL, β-cat^−/−^-ΔA, β-cat^−/−^-ΔC, and β-cat^−/−^-ΔNC cells at 24 h of PS induction. Scale bar, 100 μm. **B** Representative flow cytometry analysis of cell death in wt, β-cat^−/−^-1, β-cat^−/−^-Ctrl, β-cat^−/−^-FL, β-cat^−/−^-ΔA, β-cat^−/−^-ΔC, and β-cat^−/−^-ΔNC cells at 8 and 24 h of PS induction. **C** The summarized data from (**B**). **D** The schematic diagram of the role of β-catenin in regulating the proper PS differentiation from hPSCs. Data are represented as mean ± SEM; *n* = 3; ***p < 0.001; two-way ANOVA followed by Sidak’s post hoc.

Because the transcriptional activity of β-catenin is critical to the expression of PS genes [13, 23], we tested the hypothesis that the β-catenin-dependent cell death restriction offered a proper window for expression of lineage-specific genes during PS induction by overexpressing various truncated forms of β-catenin in β-cat^−/−^ cells. The migrated cells were only observed in the wt and β-cat^−/−^-FL cells but not in the β-cat^−/−^-ΔC and β-cat^−/−^-ΔNC cells after 24 h of induction (Fig. 7A), indicating β-cat^−/−^-ΔC cells and β-cat^−/−^-ΔNC cells cannot support the transition of hPSCs to PS lineage cells. Moreover, only the β-cat^−/−^-FL cells were able to successfully differentiate into cardiomyocytes (Video S2). β-cat^−/−^-ΔC and β-cat^−/−^-ΔNC cells died at 24 h of PS induction, though the cell death was inhibited at 8 h of PS induction (Fig. 7A–C). Taken together, these data suggest that the C-terminal region of β-catenin is critical for the cell fate transition during PS differentiation after the cells are protected from cell death by the ARM domain.

## Discussion

The in vitro differentiation system of hESCs is a valuable model in recapitulating PS formation during human development [23]. In the present study, we confirmed that hPSCs go through various stages to generate terminal-differentiated cells derived from mesendoderm, indicating the proper function of PS cells at the early induction stage. With this model, we for the first time describe a precise dynamics of cell death during human PS induction, define the novel role of β-catenin in cell death restriction during this process, and uncover the mechanism by which β-catenin inhibits cell death through the β-catenin-CK1α-DEPTOR-mTORC1-p53 axis to ensure proper PS induction. Our findings explain the observation of robust detached cells in β-catenin deficient mouse embryos [14], and suggest that the safeguard of cell survival by β-catenin supports the successful PS induction.

One of major findings is the inhibition of mTORC1 by β-catenin during PS induction. mTORC1 is essential for the maintenance of mouse inner cell mass [38–40] and hESCs [41]. Rapamycin treatment promotes the differentiation to mesendodermal cells from hESCs [41, 42], while the changes of endogenous mTORC1 activity are not fully clear. It is noticed that the culture medium used for in vitro PS differentiation lacks growth factors, such as TGFβ1, bFGF or human insulin comparing with the hPSC culture medium [43]. However, the phosphorylation level of AMPK, P38, ERK, and AKT1/2/3 are comparable between the wt and β-cat^−/−^ cells during PS induction (Fig. S8B), indicating that the inhibition of mTORC1 in the wt cells should not be the consequence of the medium changes. Supportively, the phosphorylation level of P70-S6K is lower at the PS region than that in epiblast in mouse E6.5 embryos [44]. These observations suggest that inhibiting mTORC1 is not only artificially benefiting the PS formation, but also an endogenous event during early development. Moreover, these findings link the mTORC1 inhibition with the fine-tuned developmental signals for PS formation, and suggest the versatility of WNT signaling through the β-catenin and β-catenin destruction complex acting as the “hub” for integrating the WNT signaling with other pathways in the early lineage specification.

Another finding is the identification of p53 in mediating cell death during early PS induction. Our data show the hypersensitivity of hESCs at the initial phase of differentiation relies on p53, which potentially aids in the surveillance of cell status during development and facilitates the elimination of unfit cells. However, the exact causes of cell death during early PS induction are still unclear. Peri-implantation embryos face various stresses, such as ER stress [45] and oxidative stress [46], which are also observed in ESC models [47, 48]. In addition, the gradient of various signal molecules during peri-gastrulation of early embryos might also involve into the occurrence of cell death. One possible regulator is insulin-like growth factor 1 receptor (IGF-1R) signaling, which can inhibit p53 [49]. IGF-1R signaling is critical for the survival of hESCs and mouse epiblast cells (which corresponds to hESCs). However, its inhibition is required for mesendodermal differentiation [50]. This is supported by the following evidence: (1) the IGF binding protein-4, an inhibitor of insulin/IGF-1 [51], is robustly expressed at the visceral endoderm adjacent to the PS initiation region [52]; and (2) insulin inhibits the mesendodermal differentiation from hESCs [50]. Indeed, the inhibition of IGF-1R signaling by either insulin/IGF-1 deprivation [53, 54] or PI3K/AKT inhibitors [55] is commonly used in mesendodermal differentiation protocols. We thus propose that the stresses, particularly the inhibition of IGF-1R signaling, might be the trigger for p53 activation and cell death during early PS initiation in both wt and β-cat^−/−^ cells as well as in mouse and chicken embryos [5, 8]. While β-catenin can safeguard the cells by preventing cell death from losing control during the PS induction, the exact cause for cell death in PS induction needs further investigation.

The mitochondrial translocation of P53 and mitophagy were suggested at the downstream of mTORC1 in our study, which prompts an inquiry into the potential relationship between the localization of P53 and mitochondrial autophagy. The complexity of the cross talks between p53 signaling and mitophagy has been shown [56–58]. For examples, p53 can inhibit mitophagy by interacting with Parkin in mouse hearts [56] or promote mitophagy in radioresistant cancer [57], and the initiation of mitophagy inhibits the function of mitochondrial p53 by removing the dysfunctional mitochondria [58]. These reports suggest the interaction of p53 signaling and mitophagy regulators are cell type- and condition-dependent. Our data cannot determine whether p53 promotes/inhibits mitophagy during early PS induction or the enhanced mitophagy in wild-type cells is the comprehensive outcome of the change of p53 and multiple changes (such as the inhibition of mTORC1) within the cells. More efforts should be taken to uncover the potential relationship between the p53 signaling and mitochondrial autophagy during early PS induction.

**Fig. 8:**
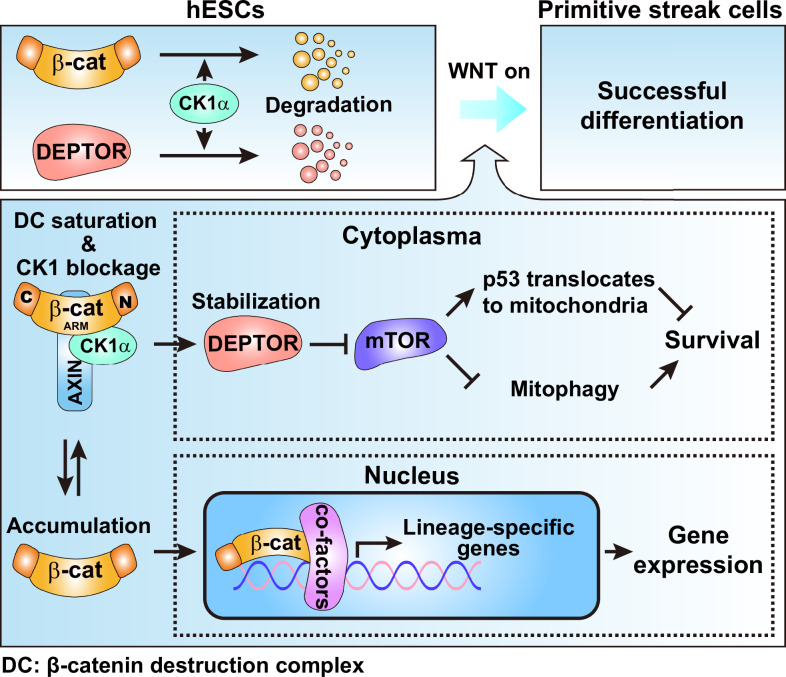
Working model of β-catenin in promoting the successful PS formation. In hESCs, CK1α promotes the degradation of β-catenin and DEPTOR to inhibit WNT signaling and maintain mTORC1 activity. During PS induction, the WNT signaling is activated. The rapidly accumulated β-catenin saturates the β-catenin destruction complex, traps CK1α into the complex, and subsequently stabilizes DEPTOR, which further inhibits mTORC1 activity. The inhibited mTORC1 results in the attenuation of mitochondrial translocation of p53 and enhancement of mitophagy, which finally promotes cell survival. Meanwhile, the downstream lineage-specific genes start to express and accumulate to reach the level for cell fate transition.

In the working model of β-catenin in promoting successful PS formation we proposed, the synergies of both transcription-independent cell death restriction and the promotion of lineage-specific genes of β-catenin safeguard the successful PS formation (Fig. 8). Although the cells with β-cat-ΔC and β-cat-ΔNC overexpression can survive after the initial period of PS induction, they cannot proceed to the functional PS cells (Fig. 7A–C). As the death of cells occurs at the first 8 h of PS induction and the main function of the C-terminal region of β-catenin is to initiate the transcription of downstream lineage-specific genes [13], we propose that the transcription-independent restriction of cell death by β-catenin serves to buffer the stress-induced cell death, thereby providing a critical time window for accumulation of β-catenin-induced lineage-specific genes to guarantee the successful fate transition of PS.

In summary, our study reveals the novel function of β-catenin in cell death restriction via interplay of WNT/β-catenin signaling, CK1α, DEPTOR, mTORC1 and p53 during human PS induction. The findings provide new insights in how β-catenin promotes PS formation and illustrate the fine-tuned coordination of differentiational signaling, cell death, and lineage transition in early fate commitment.

## Methods

### hESC culture and in vitro differentiation

hESC culture was carried out following WiCell’s instructions. Briefly, the hESC H1 and H7 lines (WiCell Research Institute) were maintained in mTeSR1 medium (Stemcell Technologies) on the Matrigel (Corning) coated dishes. Cells were passaged by ReLeSR reagent (Stemcell Technologies) following the manufacturer’s protocol.

For PS induction, hESCs was induced following a modified monolayer differentiation protocol as reported previously [59]. Briefly, hESCs were seeded onto Matrigel-coated 12-well plates at a density of 2.5 × 10^4^ cells/cm^2^ in mTeSR1 with 10 μmol/L Y-27632 (Stemcell Technologies, Vancouver, Canada) and then the medium was changed to one without Y-27632. After the hESCs reached 100% confluence, the cardiac differentiation medium (CDM3) (RPMI1640 (Gibco), 213 μg/mL l-ascorbic acid 2-phosphate (Sigma-Aldrich), 2 mg/mL bovine serum albumin (Sigma-Aldrich)) containing 6 μmol/L CHIR99021 was used to induce hESCs for 24 h. For other protocols in Fig. S3, the medium was CDM3 with indicated morphogens or small molecules. The BMP4 was used at 50 ng/ml, CHIR99021 was used at 6 μmol/L, ActinvinA was used at 100 ng/ml, WNT3a was used at 50 ng/ml.

For the cardiomyocyte differentiation, the PS-like cells are maintained with CDM3 + 6 μmol/L CHIR99021 for another 24 h for mesoderm cell induction. Then the medium was changed to CDM3 supplemented with a WNT signaling inhibitor, IWR-1 (Sigma-Aldrich) at 5 μmol/L on day 3 and day 4. In the following days, CDM3 was changed every 2 days till the beating cardiomyocytes were observed.

For neuroectodermal differentiation, we use the protocol published previously [60] with the changes in induction time. In brief, cells were grown in chemical defined medium + SB431542 10 μmol/L (Merck) + bFGF (R&D) 12 ng/ml for 4 days.

### Generation of gene knockout hESCs

The CRISPR/Cas9 technologies was used to knockout specific genes of interest in hESCs as previously reported [55]. The exon 3 was targeted for *CTNNB1* knockout and the exon 4 was targeted for *TP53* knockout. gRNAs were designed with an online tool kit “CHOPCHOP” (http://chopchop.cbu.uib.no/). The gRNA sequence and primers for sequencing were listed in Table S4. The gRNAs were constructed into pSpCas9(BB)-2A-Puro (PX459) (Plasmid #48139, Addgene) with the name PX459-gRNA-CTNNB1 and PX459-gRNA-TP53. For generate the hESCs with mutant *CTNNB1* gene, 5 μg PX459-gRNA-CTNNB1 was transfected into H1 or H7 hESCs by Nucleofection (Lonza). Forty-eight hours after nucleofection, puromycin (0.5 μg/ml) was added to remove cells without transfected plasmid. Once the cells reached 70%-80% confluency, the hESCs were digested into single cells with Accutase (Stemcell Technologies) and plated at density of 1000 cell per 10 cm dish with 10 μmol/L Y-27632 (Stemcell Technologies). Ten days later, the single cell-derived clones were picked and transferred in to 96-well plates for expansion. Sanger sequencing was used to select the clones with mutations causing early translation termination. The deficiency of β-catenin was further confirmed by western blot and immunofluorescence staining.

For generating hESCs with *TP53* knockout, the H1 and β-cat^−/−^-1 hESCs were used as the parental cells. The procedures were similar with *CTNNB1* knockout but using PX459-gRNA-TP53 plasmid.

### Lentivirus packaging

The pCDH-EF1-MCS-T2A-Puro vector (System Biosciences) was used for lentivirus packaging. Plasmids used for gene overexpression were listed in “Key resources table”. Before packaging, the plasmids were verified by Sanger sequencing, and the vector with puro overexpression was served as control plasmid. The viral package was performed with HEK293T cells (Invitrogen) after transfection of plasmids (psPAX2, pMD2.G, pCDH plasmid) with lipofectamine 2000 (Invitrogen) following the manufacturer’s instructions. 3 days after transfection, the supernatants were collected and ultracentrifuged at 130,000 × *g* for 2 h to collect the lentivirus. Lentivirus were tittered by qRT-PCR.

### Lentivirus transduction

When cells reached to the logarithmic phase, β-cat^−/−^-1 cells were transduced with indicated lentivirus containing different genes of interest at MOI = 10. Forty-eight hours after transduction, puromycin (1 μg/ml) was added to select the cells with lentivirus vectors. The cells were cultured for at least 1 week with puromycin till the morphology of colonies were normal. These cells were passaged at least 2 times before being used for each assay. The expression of transduced genes was validated by western blot and immunofluorescence staining.

### RNA interference for CSNK1A1 (the gene coding CK1α) in hESCs

The small interfering RNA (siRNA) for CSNK1A1 downregulation was designed and synthesized by Guangzhou RiboBio Co., Ltd. The transfection of siRNA to hESCs were conducted as previously reported [61]. First, 12-well plates were coated with Matrigel (Corning) and incubate at 37 °C for 1 prior to use. Next, 50 nmol/L siRNA and 2 μL DharmaFECT1 (Horizon) in were mixed with 98 μL Opti-MEM (Gibco). After removing the Matrigel, the siRNA-DharmaFECT1 mixture was transferred into each well and incubated at room temperature for 30 min. 1 × 10^6^ hESCs were suspend into 800 μL mTeSR1 containing 10 μmol/L Y-27632 and added into each well. Eighteen hours after plating, the medium was changed to normal mTeSR1.

### Immunofluorescence Staining

The immunofluorescence staining was proceed as previously reported [59]. In general, cells were fixed with 4% paraformaldehyde for 15 min, permeabilized in 0.4% Triton X-100 (Sigma-Aldrich) for 20 min at room temperature to present the intracellular antigens and blocked with 10% normal goat serum (Vector Laboratories). Antibodies were diluted into 1 × permeabilization buffer (ThermoFisher) and incubated overnight at 4 °C. After incubation, the cells were washed by 1 × permeabilization buffer for 3 times and then incubated with secondary antibodies diluted into 1×Permeabilization Buffer for 1 h at room temperature. Nuclei were stained with Hoechst (ThermoFisher) for 5 min. To detect the β-cat-FL, β-cat-ΔC, β-cat-ΔA or β-cat-ΔNC in each cell line in Fig. 5E and Fig. S7G, the anti-β-catenin antibody (BD) and anti-FLAG antibody (Sigma-Aldrich) were mixed and incubated. was used, because the anti-β-catenin antibody (BD) cannot bind β-catenin without its C-terminal domain. Images were captured on Zeiss LSM 780 confocal microscope and proceed by ZEN software.

### RNA purification

Cells were harvested after remove of the dead ones detached to the bottom of culture dishes by washing with PBS. Total RNA was extracted with an RNAprep pure Micro Kit (TIANGEN) and quantified by Nanodrop (ThermoFisher). The samples were then used for qRT-PCR or high-throughput RNA sequencing.

### Quantitative reverse transcription polymerase chain reaction (qRT-PCR)

One microgram RNA was reverse-transcribed with ReverTra Ace reverse transcriptase (Toyobo) following the manufacturer’s instructions. Quantitative PCR was carried out and analyzed by the QuantStudio™ 7 Flex Real-Time PCR System (ThermoFisher) with SYBR Green Q-PCR Master Mix (Roche). The qRT-PCR primers are listed in Table S3.

### RNA sequencing

High-throughput RNA sequencing was performed at Shanghai Majorbio Bio-pharm Biotechnology Co., Ltd. The transcriptome library was prepared following Illumina® Stranded mRNA Prep, Ligation using 1 μg of total RNA. Shortly, messenger RNA was isolated according to polyA selection method by oligo(dT) beads and then fragmented by fragmentation buffer firstly. Secondly double-stranded cDNA was synthesized using a SuperScript double-stranded cDNA synthesis kit (Invitrogen, CA) with random hexamer primers. Then the synthesized cDNA was subjected to end-repair, phosphorylation and adapter addition according to library construction protocol. Libraries were size selected for cDNA target fragments of 300 bp on 2% Low Range Ultra Agarose followed by PCR amplified using Phusion DNA polymerase (NEB) for 15 PCR cycles. After quantified by Qubit 4.0, the sequencing library was performed on HiSeq 4000 platform using HiSeq 4000 SBS Kit.

### Analysis of RNA sequencing data

The raw paired-end reads were trimmed and quality controlled by fastp [62] with default parameters. Then clean reads were separately aligned to reference genome with orientation mode using HISAT2 [63] software. The mapped reads of each sample were assembled by StringTie [64] in a reference-based approach.

To identify DEGs (differential expression genes) between two different samples, the expression level of each transcript was calculated according to the transcripts per million reads (TPM) method. RSEM [65] was used to quantify gene abundances. Essentially, differential expression analysis was performed using the DESeq2 [66]. DEGs with |log2FC| ≧ 1 and FDR < 0.05 (DESeq2) were considered to be significantly different expressed genes. In addition, functional-enrichment analysis including GO and KEGG were performed to identify which DEGs were significantly enriched in GO terms and metabolic pathways at Bonferroni-corrected *p*-value < 0.05 compared with the whole-transcriptome background. GO functional enrichment and KEGG pathway analysis were carried out by Goatools and Python scipy software, respectively.

### Western blot analysis

Cells were collected, lysed in lysis buffer to get the whole cell lysate. The cell lysate was normalized by protein concentration, which was determined by Pierce™ BCA Protein Assay Kits (ThermoFisher). Nitrocellulose membrane was used for electrotransfer and then blocked by 3% non-fat milk. Antibodies were diluted at 1:1000 ratio and incubated with the membrane overnight at 4 °C. After incubation with primary antibodies, the membranes were washed for 3 times and followed by incubation of secondary antibodies. In some conditions, the membranes were incubated together with couple of antibodies as the target bands could be distinguished by either molecular weight or the species of primary antibodies. Membranes were visualized on an Odyssey Infrared Imager (LI-COR Biosciences). Band quantification was conducted by Image Studio (LI-COR Biosciences).

### Flow cytometry analysis for intracellular antigen

The cells were handled as described previously [59], and stained with anti-CTNT antibody (Abcam, 1:1000), with PE-conjugated secondary antibody (eBioscience, 1:200) or isotype controls. The data were recorded with flow cytometry (CytoFLex LX, Beckman Coulter) and analyzed by Flowjo.

### Flow cytometry for detection of cell death

All cells including the detached and attached ones were collected into single cells. 0.1 million cells were used for 1 test. After removal of the supernatant, cells were suspended with 100 uL binding buffer (10 mmol/L Hepes, pH 7.4/140 mmol/L NaCl, 2.5 mmol/L CaCl_2_) containing 5 μl annexin V-FITC and 5 ug/ml propidium iodide (PI). The cell suspension was incubated for 15 min at room temperature. Then the percentage of dead cells was detected by flow cytometry. For data analysis, Flowjo software was used. The percentage of dead cells was calculated by the sum of percentages of cells in Annexin V-/PI+, Annexin V+/PI+, and Annexin V+/PI− populations.

### Human phospho-kinase and apoptosis array analysis

hESCs induced with PS differentiation medium for 2 h were used for human phospho-kinase array analysis by Human Phospho-Kinase Array Kit (R&D systems) following the protocol from the manufacturer. In brief, remove the differentiation medium and wash the cells with pre-cold PBS. lysis buffer 6 was added into dishes containing cells and incubate at 4 °C for 5 min with gently shaking. When cells were detached from the bottom of the plate, pipette up and down to disrupt the cells, transfer the mixture to a clean tube and incubated on ice for 30 min. Collect the supernatant of cell lysate after centrifuge at 20,000 × *g* and quantity the concentration by Bicinchoninic acid (BCA) assay. While preparing the cell lysate, block the membranes containing immobilized antibodies with blocking buffer. Dilute 200 μg protein per sample to a final volume of 2 mL with Array Buffer 1 and then incubate with each membrane overnight at 4 °C with gently shaking. For detection of phospho-kinases, incubate the membranes with detection antibody cocktail, streptavidin-HRP in sequence. For imaging, expose membranes to X-ray film for 1–10 min. After exposure, the film was scanned by high-resolution scanners. The intensity density of each spot was calculated by Image J and plotted by Graphpad.

For apoptosis array analysis, the procedure was similar with the phospho-kinase array analysis but using membranes containing antibodies against apoptosis-related protein (R&D systems).

### Co-immunoprecipitation (Co-IP) assay

Cells were harvested and lysed in Co-IP buffer (40 mmol/L Hepes [pH 7.4], 120 mmol/L NaCl, 1 mmol/L EDTA, 1% NP-40, 5% glycerol, 10 mmol/L sodium pyrophosphate, 10 mmol/L glycerol 2-phosphate, 50 mmol/L NaF, 0.5 mM sodium orthovanadate, and protease inhibitors). For Co-IP of the FLAG or HA tagged protein, the anti-FLAG or anti-HA antibody-coated magnetic beads (Sigma Alderich) were used. 10 μL beads were added into 0.5 ml lysate containing 1 mg proteins, incubated at 4 °C overnight with gently rotation. The beads with captured protein were washed with Co-IP buffer for at least 5 times. 20 μL1.5× protein loading buffer was added to the beads and incubate at 95 °C for 10 min to denature the protein. The denatured protein was examined by western blot.

For Co-IP of the tag-free protein, 1 μg antibody (anti-p53, anti-β-catenin or anti-Myc) was added to 0.5 ml lysate containing 1 mg protein and incubated at 4 °C overnight with gently rotation. After binding of antibody to the target protein, 5 μL Protein G Mag Sepharose (GE health) were added into the lysate to capture the antibody-protein complex. The beads with captured protein were washed with Co-IP buffer for at least 5 times. 15 μL 1.5× protein loading buffer was added to the beads and incubate at 95 °C for 10 min to denature the protein. The denatured protein was examined by western blot. For identify the proteins which can interact with β-catenin, the co-immunoprecipitated samples were analyzed by LC_MS in APTBIO Co., Ltd.

### Key reagents

The antibodies, small molecules, and other reagents are listed in Table S6.

### Quantification and statistical analysis

Data are represented as means ± standard error of mean (SEM). All statistical analyses were performed by the GraphPad Prism 8.0 software. In stacked bar or line plots, biological replicates and the statistics method are noted in the figure legend. Unpaired Student’s *t*-test was used when two groups were compared; one-way analysis of variance (ANOVA) followed by Tukey’s multiple comparison test was used when three or more groups were compared; two-way ANOVA followed by Sidak’s multiple comparison tests was used to compare differences between > two groups with 2 factors. *p* < 0.05 was considered statistically significant.

## Supplementary information

Supplemenraty figures

Table S1

Table S2

Table S3

Video-S1

Video-S2

Original western blot images

## Data Availability

Any additional information required to reanalyze the data reported in this paper is available from the lead contact upon request.

## References

[1] Arnold SJ, Robertson EJ. Making a commitment: cell lineage allocation and axis patterning in the early mouse embryo. Nat Rev Mol Cell Biol. 2009;10:91–103. 19129791 10.1038/nrm2618

[2] Kicheva A, Briscoe J. Control of tissue development by morphogens. Annu Rev Cell Dev Biol. 2023;39:91–121. 37418774 10.1146/annurev-cellbio-020823-011522

[3] Martyn I, Siggia ED, Brivanlou AH. Mapping cell migrations and fates in a gastruloid model to the human primitive streak. Development. 2019;146:dev179564. 10.1242/dev.179564 PMC6765127 31427289

[4] Bedzhov I, Zernicka-Goetz M. Cell death and morphogenesis during early mouse development: are they interconnected?. Bioessays. 2015;37:372–8. 25640415 10.1002/bies.201400147 PMC4409078

[5] Manova K, Tomihara-Newberger C, Wang S, Godelman A, Kalantry S, Witty-Blease K, et al. Apoptosis in mouse embryos: elevated levels in pregastrulae and in the distal anterior region of gastrulae of normal and mutant mice. Dev Dyn. 1998;213:293–308. 9825865 10.1002/(SICI)1097-0177(199811)213:3<293::AID-AJA6>3.0.CO;2-D

[6] Sanders EJ, Torkkeli PH, French AS. Patterns of cell death during gastrulation in chick and mouse embryos. Anat Embryol. 1997;195:147–54. 10.1007/s004290050033 9045984

[7] Hardy K, Handyside AH, Winston RM. The human blastocyst: cell number, death and allocation during late preimplantation development in vitro. Development. 1989;107:597–604. 2612378 10.1242/dev.107.3.597

[8] Maya-Ramos L, Mikawa T. Programmed cell death along the midline axis patterns ipsilaterality in gastrulation. Science. 2020;367:197–200. 31919222 10.1126/science.aaw2731 PMC7017582

[9] Jones SN, Roe AE, Donehower LA, Bradley A. Rescue of embryonic lethality in Mdm2-deficient mice by absence of p53. Nature. 1995;378:206–8. 7477327 10.1038/378206a0

[10] de Oca Luna Montes, Wagner R, Lozano DS. G. Rescue of early embryonic lethality in mdm2-deficient mice by deletion of p53. Nature. 1995;378:203–6. 7477326 10.1038/378203a0

[11] Shahbazi MN. Mechanisms of human embryo development: from cell fate to tissue shape and back. Development. 2020;147:dev190629. 10.1242/dev.190629 PMC7375473 32680920

[12] Clevers H, Nusse R. Wnt/beta-catenin signaling and disease. Cell. 2012;149:1192–205. 22682243 10.1016/j.cell.2012.05.012

[13] Lyashenko N, Winter M, Migliorini D, Biechele T, Moon RT, Hartmann C. Differential requirement for the dual functions of beta-catenin in embryonic stem cell self-renewal and germ layer formation. Nat Cell Biol. 2011;13:753–61. 21685890 10.1038/ncb2260 PMC3130149

[14] Haegel H, Larue L, Ohsugi M, Fedorov L, Herrenknecht K, Kemler R. Lack of beta-catenin affects mouse development at gastrulation. Development. 1995;121:3529–37. 8582267 10.1242/dev.121.11.3529

[15] Knippschild U, Kruger M, Richter J, Xu P, Garcia-Reyes B, Peifer C, et al. The CK1 family: contribution to cellular stress response and its role in carcinogenesis. Front Oncol. 2014;4:96. 24904820 10.3389/fonc.2014.00096 PMC4032983

[16] Burridge PW, Matsa E, Shukla P, Lin ZC, Churko JM, Ebert AD, et al. Chemically defined generation of human cardiomyocytes. Nat Methods. 2014;11:855–60. 24930130 10.1038/nmeth.2999 PMC4169698

[17] Tovar C, Rosinski J, Filipovic Z, Higgins B, Kolinsky K, Hilton H, et al. Small-molecule MDM2 antagonists reveal aberrant p53 signaling in cancer: implications for therapy. Proc Natl Acad Sci USA. 2006;103:1888–93. 16443686 10.1073/pnas.0507493103 PMC1413632

[18] Zhang X, Li CF, Zhang L, Wu CY, Han L, Jin G, et al. TRAF6 Restricts p53 Mitochondrial translocation, apoptosis, and tumor suppression. Mol Cell. 2016;64:803–14. 27818144 10.1016/j.molcel.2016.10.002 PMC5541903

[19] Moll UM, Wolff S, Speidel D, Deppert W. Transcription-independent pro-apoptotic functions of p53. Curr Opin Cell Biol. 2005;17:631–6. 16226451 10.1016/j.ceb.2005.09.007

[20] Chipuk JE, Kuwana T, Bouchier-Hayes L, Droin NM, Newmeyer DD, Schuler M, et al. Direct activation of Bax by p53 mediates mitochondrial membrane permeabilization and apoptosis. Science. 2004;303:1010–4. 14963330 10.1126/science.1092734

[21] Strom E, Sathe S, Komarov PG, Chernova OB, Pavlovska I, Shyshynova I, et al. Small-molecule inhibitor of p53 binding to mitochondria protects mice from gamma radiation. Nat Chem Biol. 2006;2:474–9. 16862141 10.1038/nchembio809

[22] Komarov PG, Komarova EA, Kondratov RV, Christov-Tselkov K, Coon JS, Chernov MV, et al. A chemical inhibitor of p53 that protects mice from the side effects of cancer therapy. Science. 1999;285:1733–7. 10481009 10.1126/science.285.5434.1733

[23] Funa NS, Schachter KA, Lerdrup M, Ekberg J, Hess K, Dietrich N, et al. beta-Catenin Regulates Primitive Streak Induction through Collaborative Interactions with SMAD2/SMAD3 and OCT4. Cell Stem Cell. 2015;16:639–52. 25921273 10.1016/j.stem.2015.03.008

[24] Kruse JP, Gu W. Modes of p53 regulation. Cell. 2009;137:609–22. 19450511 10.1016/j.cell.2009.04.050 PMC3737742

[25] Hornberger TA, Sukhija KB, Wang XR, Chien S. mTOR is the rapamycin-sensitive kinase that confers mechanically-induced phosphorylation of the hydrophobic motif site Thr(389) in p70(S6k). FEBS Lett. 2007;581:4562–6. 17825298 10.1016/j.febslet.2007.08.045 PMC2084087

[26] Kim JH, Kim JE, Liu HY, Cao W, Chen J. Regulation of interleukin-6-induced hepatic insulin resistance by mammalian target of rapamycin through the STAT3-SOCS3 pathway. J Biol Chem. 2008;283:708–15. 17993646 10.1074/jbc.M708568200

[27] Kim JH, Yoon MS, Chen J. Signal transducer and activator of transcription 3 (STAT3) mediates amino acid inhibition of insulin signaling through serine 727 phosphorylation. J Biol Chem. 2009;284:35425–32. 19875458 10.1074/jbc.M109.051516 PMC2790971

[28] Yu GT, Bu LL, Zhao YY, Liu B, Zhang WF, Zhao YF, et al. Inhibition of mTOR reduce Stat3 and PAI related angiogenesis in salivary gland adenoid cystic carcinoma. Am J Cancer Res. 2014;4:764–75. 25520866 PMC4266710

[29] Gu S, Tan J, Li Q, Liu S, Ma J, Zheng Y, et al. Downregulation of LAPTM4B contributes to the impairment of the autophagic flux via unopposed activation of mTORC1 signaling during myocardial ischemia/reperfusion injury. Circ Res. 2020;127:e148–e165. 32693673 10.1161/CIRCRESAHA.119.316388

[30] Bin-Umer MA, McLaughlin JE, Butterly MS, McCormick S, Tumer NE. Elimination of damaged mitochondria through mitophagy reduces mitochondrial oxidative stress and increases tolerance to trichothecenes. Proc Natl Acad Sci USA. 2014;111:11798–803. 25071194 10.1073/pnas.1403145111 PMC4136610

[31] Bartolome A, Garcia-Aguilar A, Asahara SI, Kido Y, Guillen C, Pajvani UB, et al. MTORC1 regulates both general autophagy and mitophagy induction after oxidative phosphorylation uncoupling. Mol Cell Biol. 2017;37:e00441–17. 10.1128/MCB.00441-17 PMC5686580 28894028

[32] Berezhnov AV, Soutar MP, Fedotova EI, Frolova MS, Plun-Favreau H, Zinchenko VP, et al. Intracellular pH modulates autophagy and mitophagy. J Biol Chem. 2016;291:8701–8. 26893374 10.1074/jbc.M115.691774 PMC4861439

[33] Peterson TR, Laplante M, Thoreen CC, Sancak Y, Kang SA, Kuehl WM, et al. DEPTOR is an mTOR inhibitor frequently overexpressed in multiple myeloma cells and required for their survival. Cell. 2009;137:873–86. 19446321 10.1016/j.cell.2009.03.046 PMC2758791

[34] Zhao Y, Xiong X, Sun Y. DEPTOR, an mTOR inhibitor, is a physiological substrate of SCF(betaTrCP) E3 ubiquitin ligase and regulates survival and autophagy. Mol Cell. 2011;44:304–16. 22017876 10.1016/j.molcel.2011.08.029 PMC3216641

[35] Duan S, Skaar JR, Kuchay S, Toschi A, Kanarek N, Ben-Neriah Y, et al. mTOR generates an auto-amplification loop by triggering the betaTrCP- and CK1alpha-dependent degradation of DEPTOR. Mol Cell. 2011;44:317–24. 22017877 10.1016/j.molcel.2011.09.005 PMC3212871

[36] Xu W, Kimelman D. Mechanistic insights from structural studies of beta-catenin and its binding partners. J Cell Sci. 2007;120:3337–44. 17881495 10.1242/jcs.013771

[37] Romagnolo B, Berrebi D, Saadi-Keddoucci S, Porteu A, Pichard AL, Peuchmaur M, et al. Intestinal dysplasia and adenoma in transgenic mice after overexpression of an activated beta-catenin. Cancer Res. 1999;59:3875–9. 10463573

[38] Gangloff YG, Mueller M, Dann SG, Svoboda P, Sticker M, Spetz JF, et al. Disruption of the mouse mTOR gene leads to early postimplantation lethality and prohibits embryonic stem cell development. Mol Cell Biol. 2004;24:9508–16. 15485918 10.1128/MCB.24.21.9508-9516.2004 PMC522282

[39] Murakami M, Ichisaka T, Maeda M, Oshiro N, Hara K, Edenhofer F, et al. mTOR is essential for growth and proliferation in early mouse embryos and embryonic stem cells. Mol Cell Biol. 2004;24:6710–8. 15254238 10.1128/MCB.24.15.6710-6718.2004 PMC444840

[40] Guertin DA, Stevens DM, Thoreen CC, Burds AA, Kalaany NY, Moffat J, et al. Ablation in mice of the mTORC components raptor, rictor, or mLST8 reveals that mTORC2 is required for signaling to Akt-FOXO and PKCalpha, but not S6K1. Dev Cell. 2006;11:859–71. 17141160 10.1016/j.devcel.2006.10.007

[41] Zhou J, Su P, Wang L, Chen J, Zimmermann M, Genbacev O, et al. mTOR supports long-term self-renewal and suppresses mesoderm and endoderm activities of human embryonic stem cells. Proc Natl Acad Sci USA. 2009;106:7840–5. 19416884 10.1073/pnas.0901854106 PMC2683106

[42] Nazareth EJP, Rahman N, Yin T, Zandstra PW. A multi-lineage screen reveals mtorc1 inhibition enhances human pluripotent stem cell mesendoderm and blood progenitor production. Stem Cell Rep. 2016;6:679–91. 10.1016/j.stemcr.2016.04.003 PMC4939733 27132889

[43] Ludwig TE, Levenstein ME, Jones JM, Berggren WT, Mitchen ER, Frane JL, et al. Derivation of human embryonic stem cells in defined conditions. Nat Biotechnol. 2006;24:185–7. 16388305 10.1038/nbt1177

[44] Bowling S, Di Gregorio A, Sancho M, Pozzi S, Aarts M, Signore M, et al. P53 and mTOR signalling determine fitness selection through cell competition during early mouse embryonic development. Nat Commun. 2018;9:1763. 29720666 10.1038/s41467-018-04167-y PMC5932021

[45] Michalak M, Gye MC. Endoplasmic reticulum stress in periimplantation embryos. Clin Exp Reprod Med. 2015;42:1–7. 25874167 10.5653/cerm.2015.42.1.1 PMC4390675

[46] Deluao JC, Winstanley Y, Robker RL, Pacella-Ince L, Gonzalez MB, McPherson NO. OXIDATIVE STRESS AND REPRODUCTIVE FUNCTION: Reactive oxygen species in the mammalian pre-implantation embryo. Reproduction. 2022;164:F95–F108. 36111646 10.1530/REP-22-0121

[47] Kim JS, Hwang SI, Ryu JL, Hong HS, Lee JM, Lee SM, et al. ER stress reliever enhances functionalities of in vitro cultured hepatocytes. Stem Cell Res. 2020;43:101732. 32087526 10.1016/j.scr.2020.101732

[48] Hu Q, Khanna P, Ee Wong BS, Lin Heng ZS, Subhramanyam CS, Thanga LZ, et al. Oxidative stress promotes exit from the stem cell state and spontaneous neuronal differentiation. Oncotarget. 2018;9:4223–38. 29423117 10.18632/oncotarget.23786 PMC5790534

[49] Werner H, Sarfstein R, LeRoith D, Bruchim I. Insulin-like growth factor 1 signaling axis meets p53 genome protection pathways. Front Oncol. 2016;6:159. 27446805 10.3389/fonc.2016.00159 PMC4917523

[50] Freund C, Ward-van Oostwaard D, Monshouwer-Kloots J, van den Brink S, van Rooijen M, Xu X, et al. Insulin redirects differentiation from cardiogenic mesoderm and endoderm to neuroectoderm in differentiating human embryonic stem cells. Stem Cells. 2008;26:724–33. 18096723 10.1634/stemcells.2007-0617

[51] Contois LW, Nugent DP, Caron JM, Cretu A, Tweedie E, Akalu A, et al. Insulin-like growth factor binding protein-4 differentially inhibits growth factor-induced angiogenesis. J Biol Chem. 2012;287:1779–89. 22134921 10.1074/jbc.M111.267732 PMC3265860

[52] Wang R, Yang X, Chen J, Zhang L, Griffiths JA, Cui G, et al. Time space and single-cell resolved tissue lineage trajectories and laterality of body plan at gastrulation. Nat Commun. 2023;14:5675. 37709743 10.1038/s41467-023-41482-5 PMC10502153

[53] Lian X, Hsiao C, Wilson G, Zhu K, Hazeltine LB, Azarin SM, et al. Robust cardiomyocyte differentiation from human pluripotent stem cells via temporal modulation of canonical Wnt signaling. Proc Natl Acad Sci USA. 2012;109:E1848–1857. 22645348 10.1073/pnas.1200250109 PMC3390875

[54] Oldershaw RA, Baxter MA, Lowe ET, Bates N, Grady LM, Soncin F, et al. Directed differentiation of human embryonic stem cells toward chondrocytes. Nat Biotechnol. 2010;28:1187–94. 20967028 10.1038/nbt.1683

[55] Liang H, Zhang P, Bai HJ, Huang J, Yang HT. TATA box-binding protein-related factor 3 drives the mesendoderm specification of human embryonic stem cells by globally interacting with the TATA box of key mesendodermal genes. Stem Cell Res Ther. 2020;11:196. 32448362 10.1186/s13287-020-01711-w PMC7245780

[56] Hoshino A, Mita Y, Okawa Y, Ariyoshi M, Iwai-Kanai E, Ueyama T, et al. Cytosolic p53 inhibits Parkin-mediated mitophagy and promotes mitochondrial dysfunction in the mouse heart. Nat Commun. 2013;4:2308. 23917356 10.1038/ncomms3308

[57] Chang HW, Kim MR, Lee HJ, Lee HM, Kim GC, Lee YS, et al. p53/BNIP3-dependent mitophagy limits glycolytic shift in radioresistant cancer. Oncogene. 2019;38:3729–42. 30664690 10.1038/s41388-019-0697-6

[58] Liu K, Lee J, Kim JY, Wang L, Tian Y, Chan ST, et al. Mitophagy controls the activities of tumor suppressor p53 to regulate hepatic cancer stem cells. Mol Cell. 2017;68:281–292.e285. 29033320 10.1016/j.molcel.2017.09.022 PMC5687282

[59] Zhang P, Huang JJ, Ou-Yang KF, Liang H, Li ML, Wang YJ, et al. Minimal contribution of IP3R2 in cardiac differentiation and derived ventricular-like myocytes from human embryonic stem cells. Acta Pharm Sin. 2020;41:1576–86. 10.1038/s41401-020-00528-w PMC7921119 33037404

[60] Bai HJ, Zhang P, Ma L, Liang H, Wei G, Yang HT. SMYD2 Drives Mesendodermal differentiation of human embryonic stem cells through mediating the transcriptional activation of key mesendodermal genes. Stem Cells. 2019;37:1401–15. 31348575 10.1002/stem.3068

[61] Hu JL, Liang H, Zhang H, Yang MZ, Sun W, Zhang P, et al. FAM46B is a prokaryotic-like cytoplasmic poly(A) polymerase essential in human embryonic stem cells. Nucleic Acids Res. 2020;48:2733–48. 32009146 10.1093/nar/gkaa049 PMC7049688

[62] Chen S, Zhou Y, Chen Y, Gu J. fastp: an ultra-fast all-in-one FASTQ preprocessor. Bioinformatics. 2018;34:i884–i890. 30423086 10.1093/bioinformatics/bty560 PMC6129281

[63] Kim D, Paggi JM, Park C, Bennett C, Salzberg SL. Graph-based genome alignment and genotyping with HISAT2 and HISAT-genotype. Nat Biotechnol. 2019;37:907–15. 31375807 10.1038/s41587-019-0201-4 PMC7605509

[64] Pertea M, Pertea GM, Antonescu CM, Chang TC, Mendell JT, Salzberg SL. StringTie enables improved reconstruction of a transcriptome from RNA-seq reads. Nat Biotechnol. 2015;33:290–5. 25690850 10.1038/nbt.3122 PMC4643835

[65] Li B, Dewey CN. RSEM: accurate transcript quantification from RNA-Seq data with or without a reference genome. BMC Bioinform. 2011;12:323. 10.1186/1471-2105-12-323 PMC3163565 21816040

[66] Love MI, Huber W, Anders S. Moderated estimation of fold change and dispersion for RNA-seq data with DESeq2. Genome Biol. 2014;15:550. 25516281 10.1186/s13059-014-0550-8 PMC4302049

