# Supplementary material for: β-catenin safeguards cell survival via a transcription-independent mechanism during the induction of primitive streak from hESCs
Source: Cell Death Discov. 2025 Jul 2;11:300. doi: 10.1038/s41420-025-02559-w (PMC12222672; doi:10.1038/s41420-025-02559-w)
Supplement: Supplementary file 1 — Supplemenraty figures [file 41420_2025_2559_MOESM1_ESM.docx]

**Supplementary data for**

**β-catenin safeguards cells against death via a transcription-independent mechanism during the induction of primitive streak from hESCs**

**Peng Zhang, Xu-xia Li, Hua-Jun Bai, Yongxu Zhao, Senle Rao, He Liang, Xiao-Ling Luo, and Huang-Tian Yang**

**This file contains**

| Supplementary figure S1-S10 |
| --- |
| Legend of supplementary table S1-S3 |
| Supplementary table S4-S6 |
| Legend of Supplementary video S1-S2 |

**Supplementary figures**

**
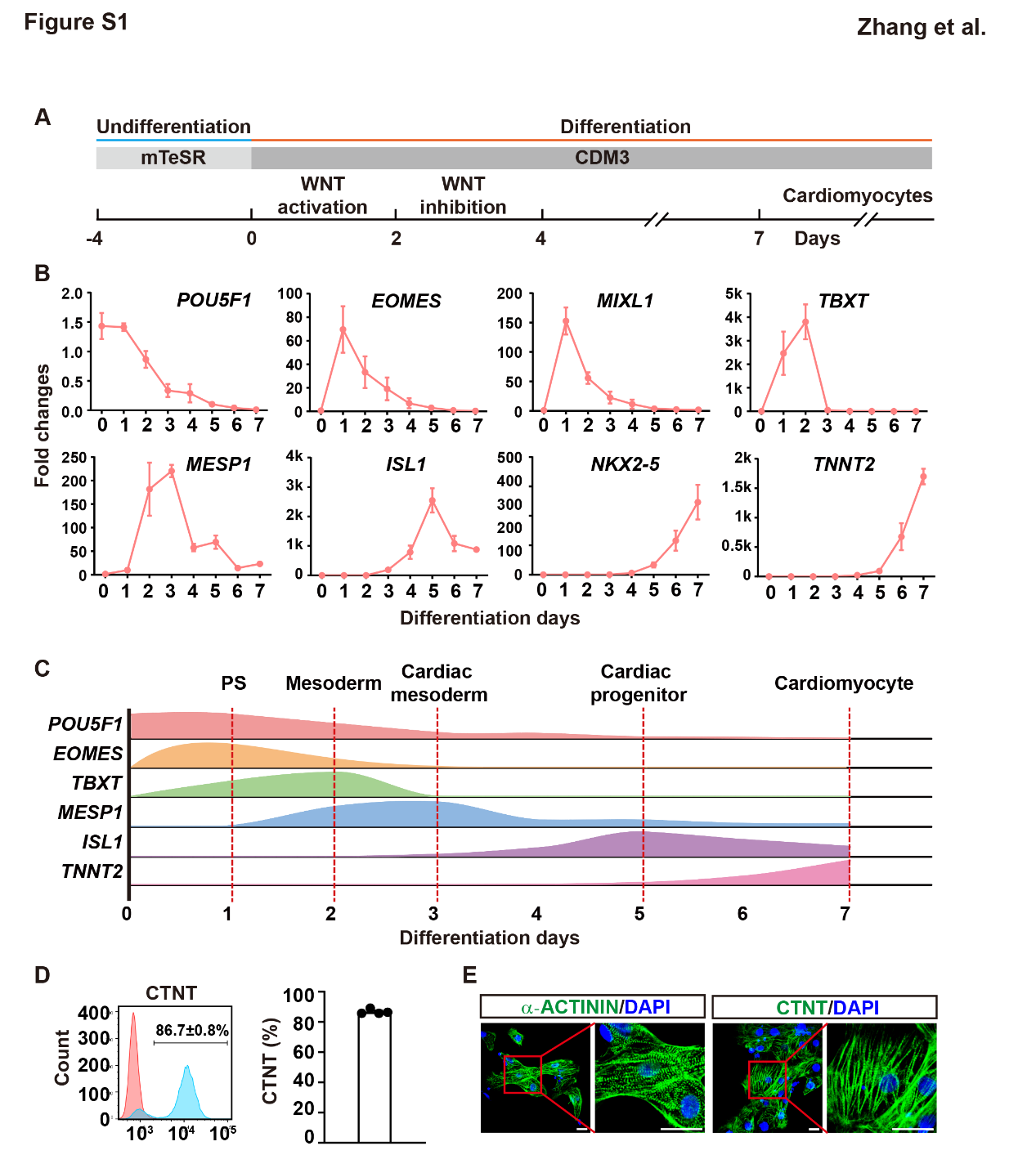
**

**Figure S1. Validation of PS induction method from hESCs, related to Figure 1.**

(**A**) The schematic diagram of the differentiation protocol from hESCs to cardiomyocytes.

(**B**) qRT-PCR analysis of mRNA expression of genes during cardiac differentiation from H1 hESCs. *POU5F1*, pluripotency marker gene; *EOMES*, *MIXL1*, PS marker genes; *TBXT*, mesoderm marker genes; *MESP1*, cardiac mesoderm marker gene; *ISL1, MEF2C* and *NKX2-5*, cardiac progenitor marker genes; *TNNT2*, cardiomyocyte marker. Data are represented as mean ± SEM; *n* = 3.

(**C**) The sequential stages during the differentiation from hESCs to cardiomyocytes based on the expression profile of stage marker genes in B.

(**D**) Representative (left panel) and summarized data (right panel) of Flow cytometry analysis for detecting the cardiomyocyte marker CTNT in the cardiomyocytes at differentiation day 14.

(**E**) Immunocytochemical staining of the cardiomyocytes at differentiation day 90 with a sarcomeric marker ɑ-ACTININ. Scale bar, 20 μm.


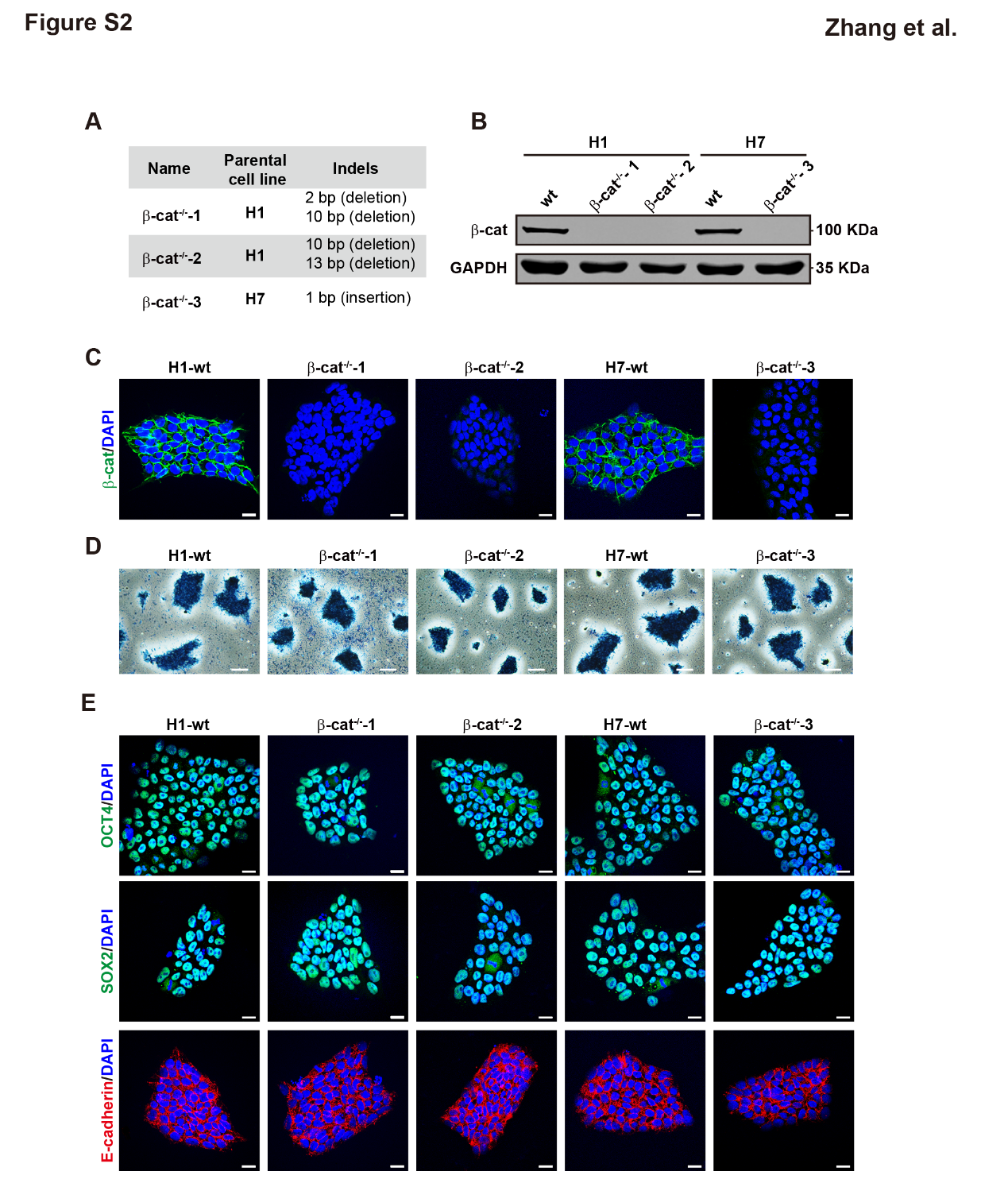


**Figure S2. The generation and characterization of β-catenin deficient (β-cat^-/-^) hESCs, related to Figure 1.**

(**A**) The name and the indels of each clone.

(**B**) Western blot analysis of each β-cat^-/-^ hESCs.

(**C**) Immunofluorescence staining of β-catenin in parental and β-cat^-/-^ hESCs. wt, wild type H1 cells; β-cat^-/-^-1, β-catenin deficient cells Clone 1; β-cat^-/-^-Ctrl, β-cat^-/-^-1 cells infected with the control lentivirus; β-cat^-/-^-FL, β-cat^-/-^-1 cells infected with lentivirus containing full-length β-catenin. Scale bar, 20 μm.

(**D**) The ALP staining in parental and β-cat^-/-^ hESCs. Scale bar, 100 μm.

(**E**) Immunofluorescence staining of pluripotent markers in parental and β-cat^-/-^ hESCs. Scale bar, 20 μm.


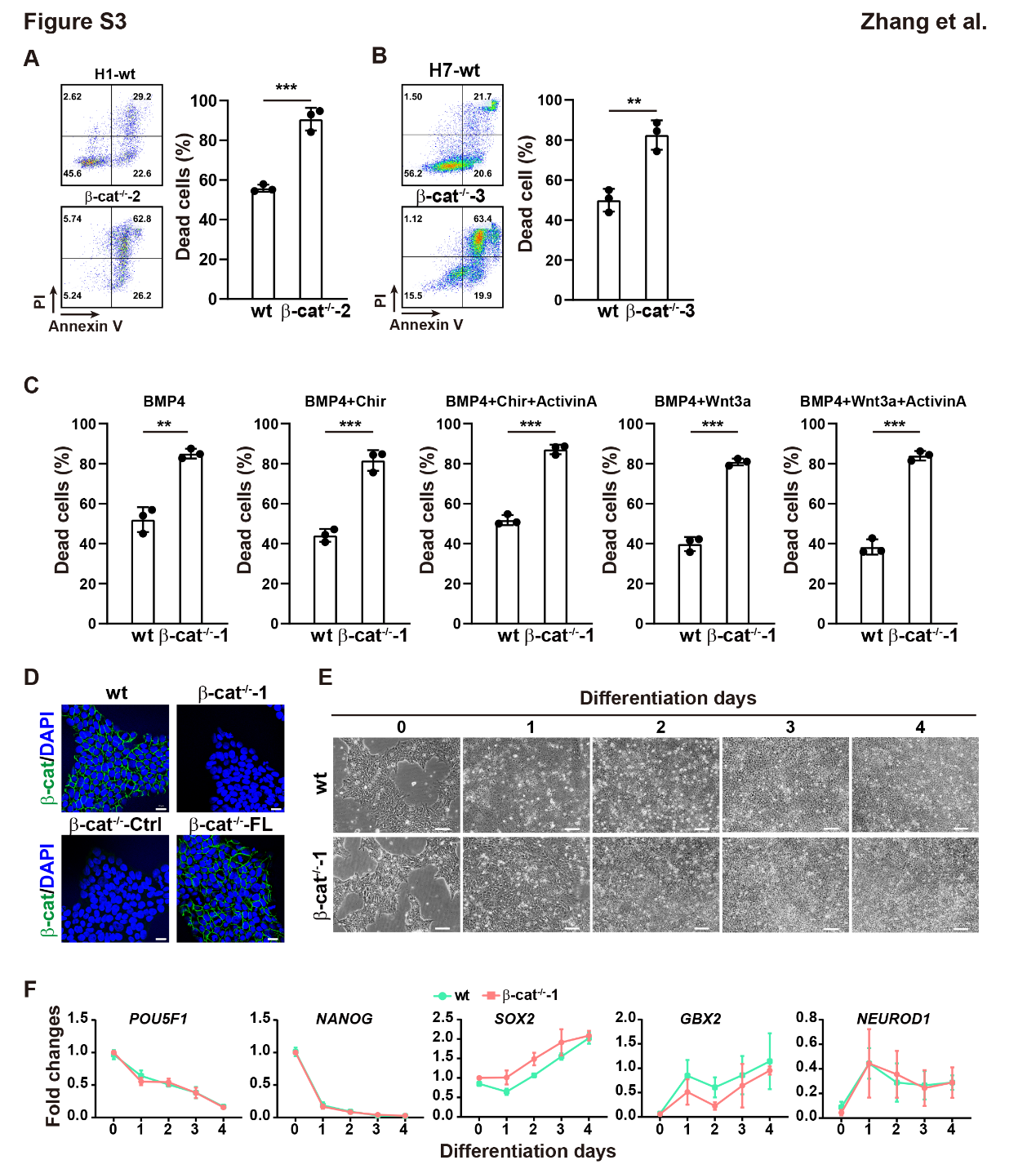


**Figure S3. Cell death detection in various conditions, related to Figure 1.**

(**A**) - (**B**) Flow cytometry analysis of β-cat^-/-^-2 (**A**) and β-cat^-/-^-3 (**B**) hESCs at 8 hours after PS induction. The β-cat^-/-^-2 hESC line is derived from H1, and the β-cat^-/-^-3 hESCs is generated from the H7. Data are represented as mean ± SEM; *n* = 3; **p < 0.01, ***p < 0.001; Student’s t test.

(**C**) Flow cytometry analysis of cell death of β-cat^-/-^-1 hESCs at 8 hours of PS induction by using different PS or mesendodermal differentiation protocols. Data are represented as mean ± SEM; *n* = 3; **p < 0.01, ***p < 0.001; Student’s t test.

(**D**) Immunofluorescence staining of β-catenin in each hESC lines. Scale bar, 20 μm.

(**E**) Morphology of wt and β-cat^-/-^-1 cells during neuroectodermal differentiation. Scale bar, 100 μm.

(**F**) qRT-PCR analysis of the expression of pluripotent markers genes (*POU5F1*, *NANOG*) and neuroectodermal marker genes (*SOX2*, *GBX2*, and *NEUROD1*).


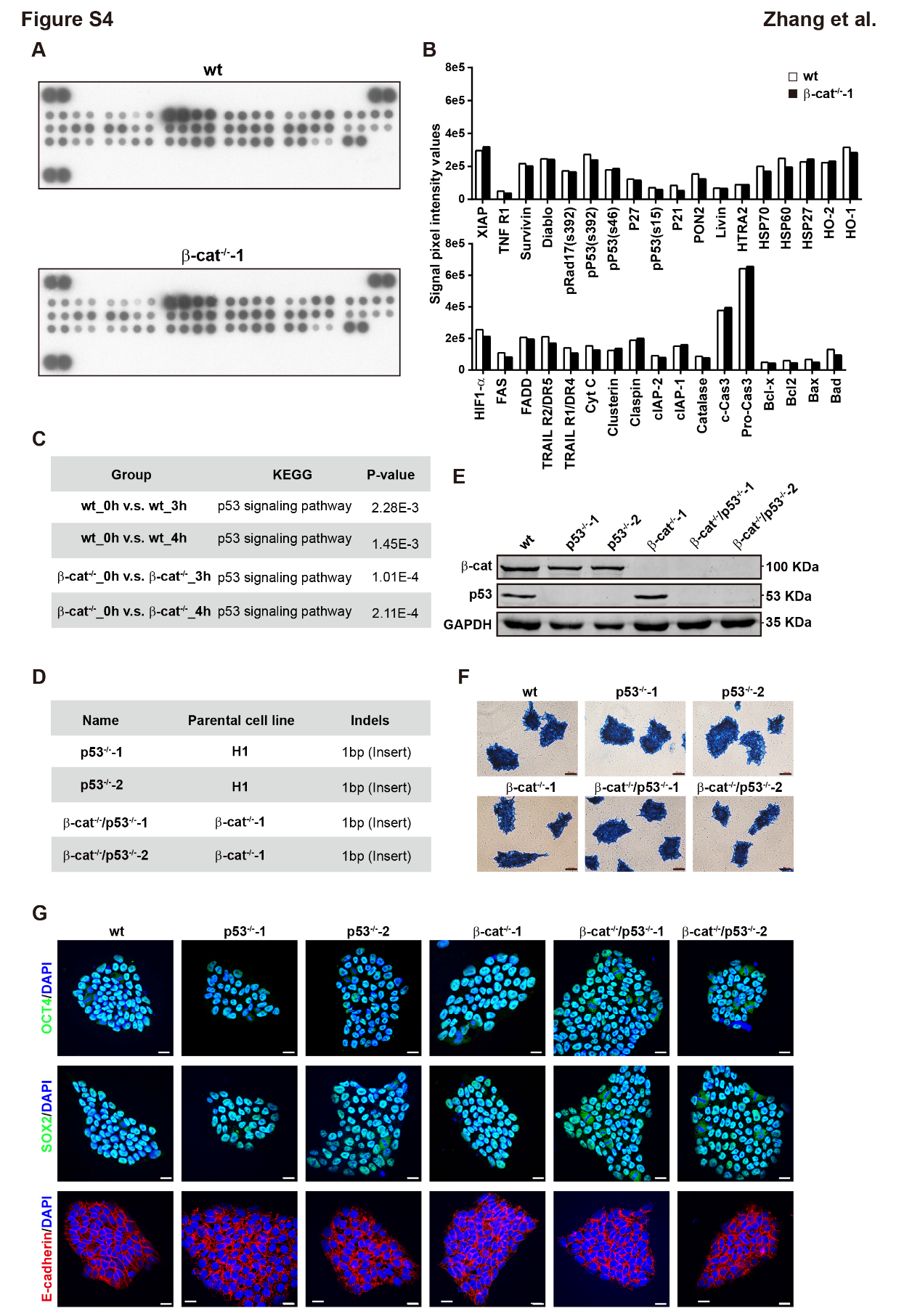


**Figure S4. The apoptotic array assay, KEGG enrichment and characterization of p53 deficient hESCs, related to Figure 2.**

**(A**) The apoptotic antibody array analysis of wt and β-cat^-/-^-1 cells after 2 hours of PS induction.

**(B**) The mean signal pixel intensity values of each protein.

(**C**) p53 signaling pathway was significantly enriched in DEGs at 3 and 4 hours after PS induction in both wt and β-cat^-/-^-1 cells.

(**D**) The name and the indels of each clone.

(**E**) Western blot analysis of the expression of β-catenin and p53 in each hESC line.

(**F**) The ALP staining in each hESC line. Scale bar, 100 μm.

(**G**) Immunocytochemical staining of pluripotent markers in each hESC line. Scale bar, 20 μm.

**
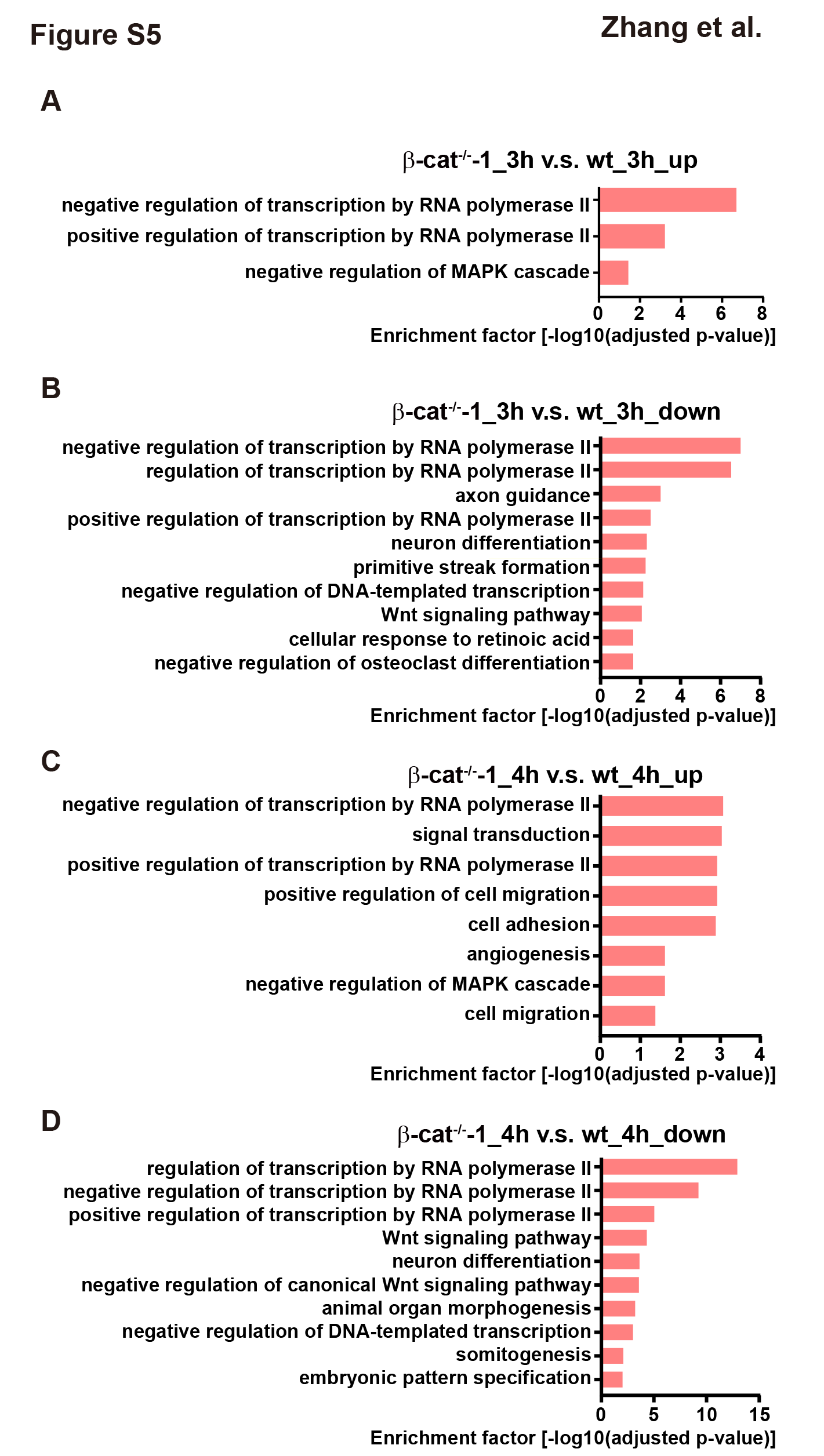
**

**Figure S5. The Gene Ontology (GO) term enrichment assay of DEGs between the wt and β-cat^-/-^-1 at 3 and 4 hours after differentiation.**

**(A**) The enriched biological processes by analyzing the upregulated DEGs in the β-cat^-/-^-1 cells comparing with wt cells after 3 hours of differentiation.

**(B**) The enriched biological processes by analyzing the downregulated DEGs in β-cat^-/-^-1 cells comparing with wt cells after 3 hours of differentiation.

**(C**) The enriched biological processes by analyzing the upregulated DEGs in β-cat^-/-^-1 cells comparing with wt cells after 4 hours of differentiation.

**(D**) The enriched biological processes by analyzing the downregulated DEGs in β-cat^-/-^-1 cells comparing with wt cells after 4 hours of differentiation.


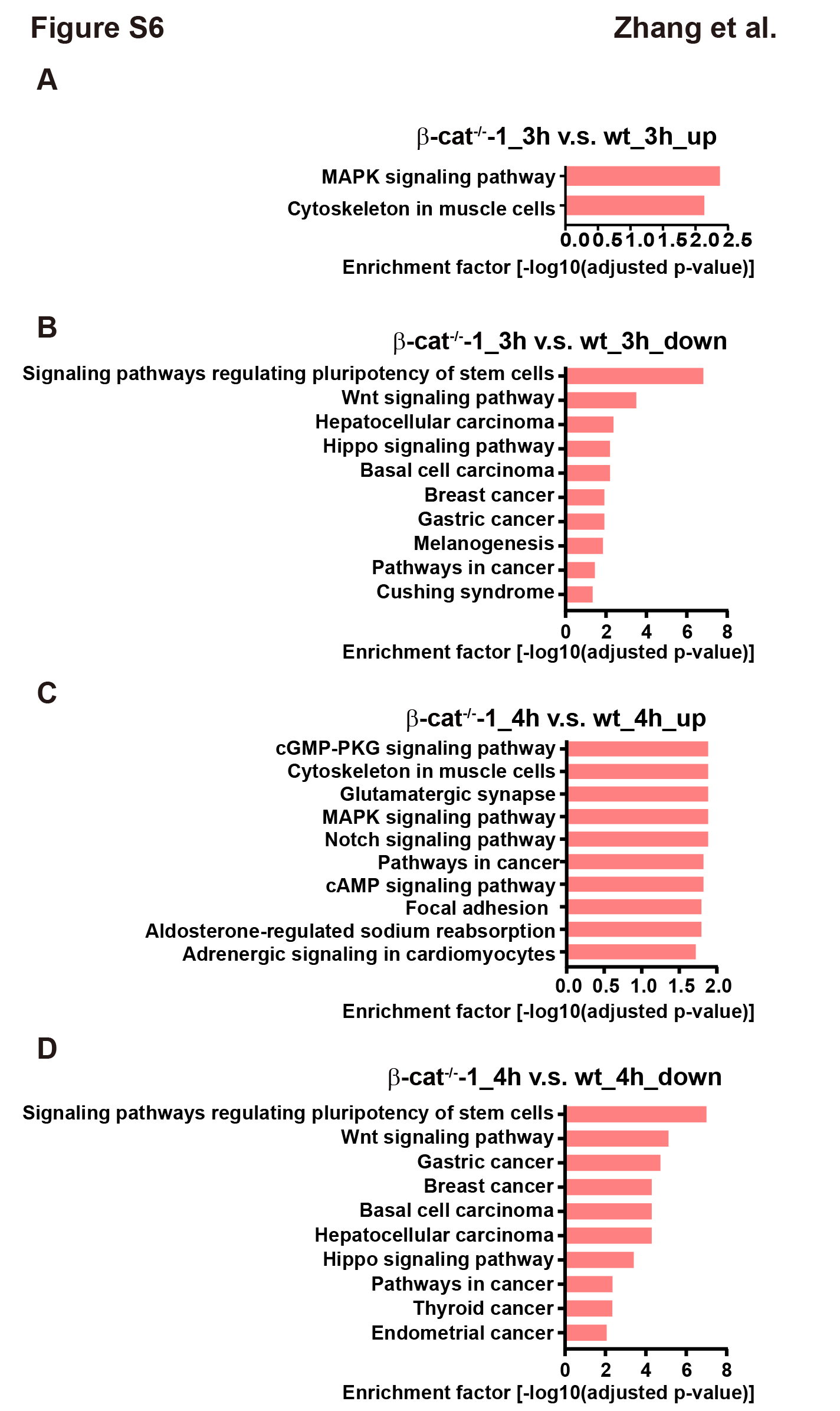


**Figure S6. The KEGG pathway enrichment assay of DEGs between wt and β-cat^-/-^-1 at 3 and 4 hours after differentiation.**

**(A**) The enriched pathways by analyzing the upregulated DEGs in β-cat^-/-^-1 cells comparing with wt cells after 3 hours of differentiation.

**(B**) The enriched pathways by analyzing the downregulated DEGs in β-cat^-/-^-1 cells comparing with wt cells after 3 hours of differentiation.

**(C**) The enriched pathways by analyzing the upregulated DEGs in β-cat^-/-^-1 cells comparing with wt cells after 4 hours of differentiation.

**(D**) The enriched pathways by analyzing the downregulated DEGs in β-cat^-/-^-1 cells comparing with wt cells after 4 hours of differentiation.


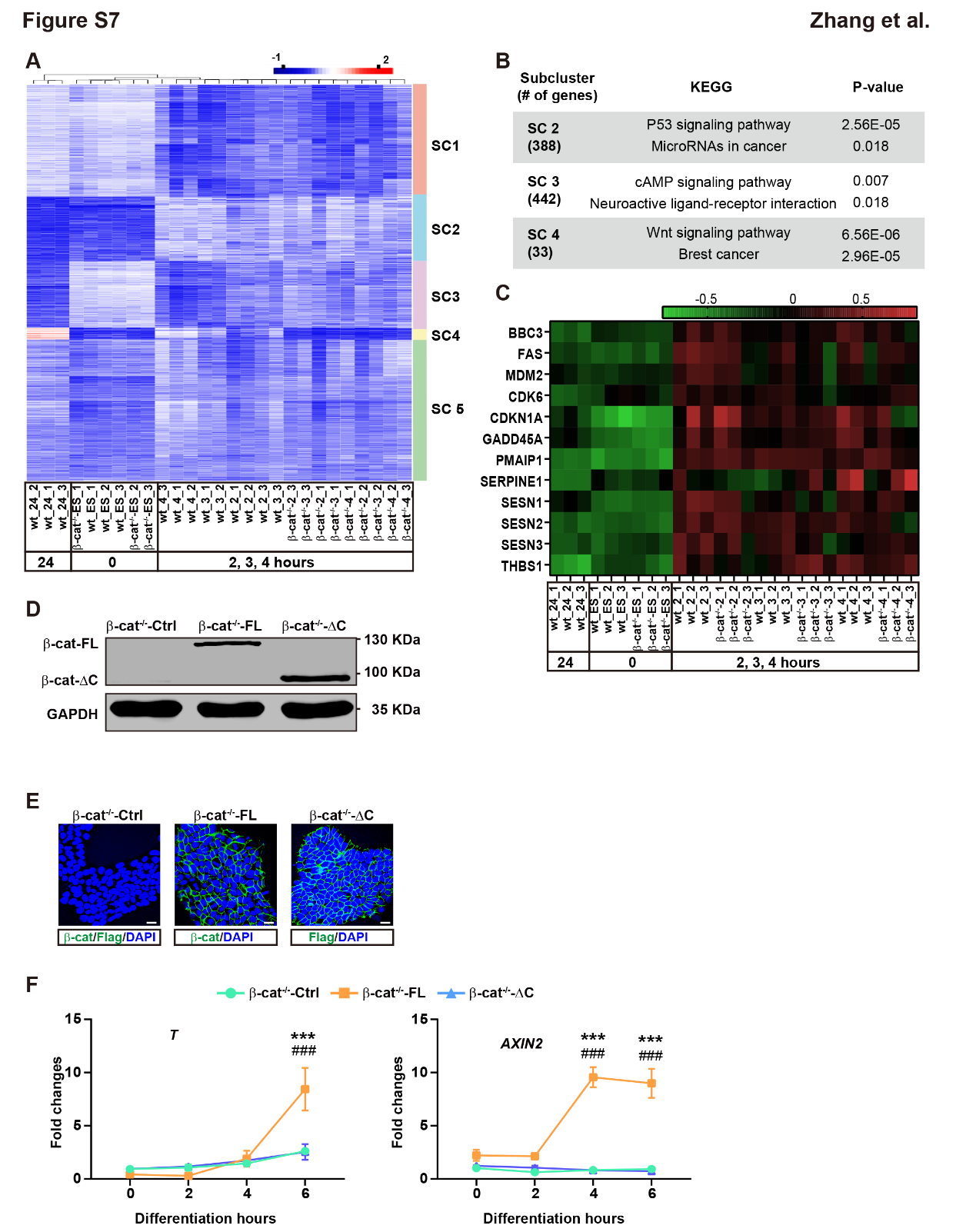


**Figure S7. The cell death restriction effect of β-catenin during early PS differentiation is transcriptional-independent, related to Figure 3.**

(**A**) Heatmap showing log10 (TPM+1) of DEGs. The DEGs by comparing the expression of genes in cells at 2, 3, 4 hours with the genes of cells at undifferentiated state in both wt and β-cat^-/-^-1 hESCs were gathered for clustering. The sub-clusters (SC) were indicated on the right of the heatmap.

(**B**) KEGG analysis of DEGs in each subclusters in A. The top 2 pathways with p < 0.05 in each SCs were shown. No pathways in SC-1 and SC-4 were statistically enriched.

(**C**) Heatmap of DEGs with key words “apoptotic” revealed by GO analysis of DEGs in A. A total number of 76 genes were included and were proceeded by clustering.

(**D**) Validation of the expression of different forms of β-catenin in each hESC line by western blot. β-cat^-/-^-ΔC, β-cat^-/-^-1 cells infected with the lentivirus containing deleted C-terminal domain of β-catenin (β-cat-ΔC). FLAG is fused to the N terminal of β-cat-ΔC for detection. GAPDH is used as loading control.

(**E**) Immunocytochemical staining of different forms of β-catenin in each hESCs. The β-catenin with C-terminal deletion (ΔC) is fused with FLAG tag for detection. Scale bar, 20 μm.

(**F**) qRT-PCR analysis of the downstream genes of β-catenin in each hESCs after PS induction. Data are represented as mean ± SEM; *n* = 3; ***p < 0.001 *vs.*β-cat^-/-^-Ctrl cells; ^###^p < 0.001 *vs.*β-cat^-/-^-ΔC cells; two-way ANOVA followed by Sidak’s post hoc.


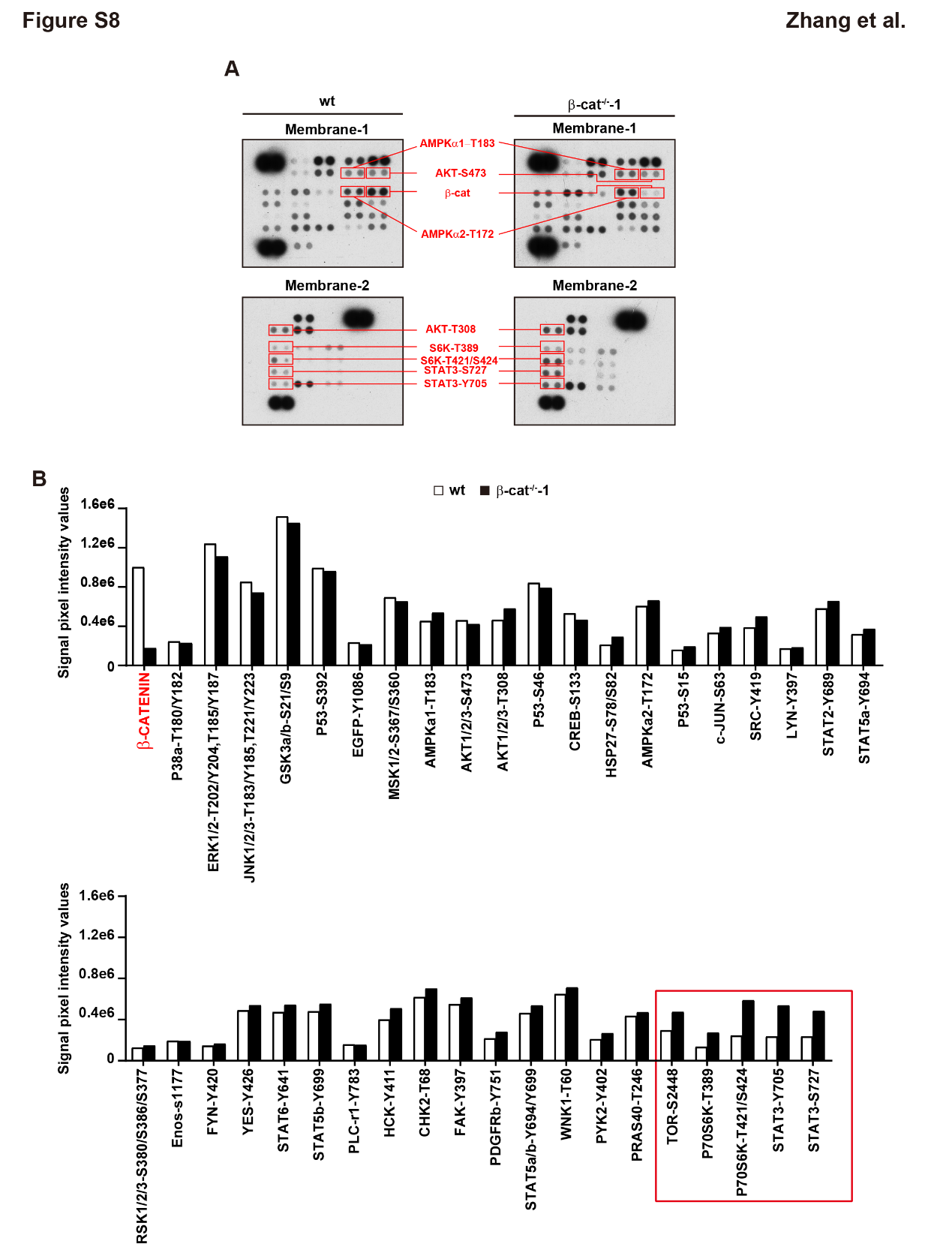


**Figure S8. Antibody array analysis revealed the abnormal activation of mTOR signaling, related to Figure 4.**

**(A**) The protein kinase antibody array analysis of wt and β-cat^-/-^-1 cells after 2 hours of PS induction. The key proteins related to mTOR signaling is marked by red box.

**(B**) The mean signal pixel intensity values of each protein. Red box indicated the proteins with fold change over 1.5.


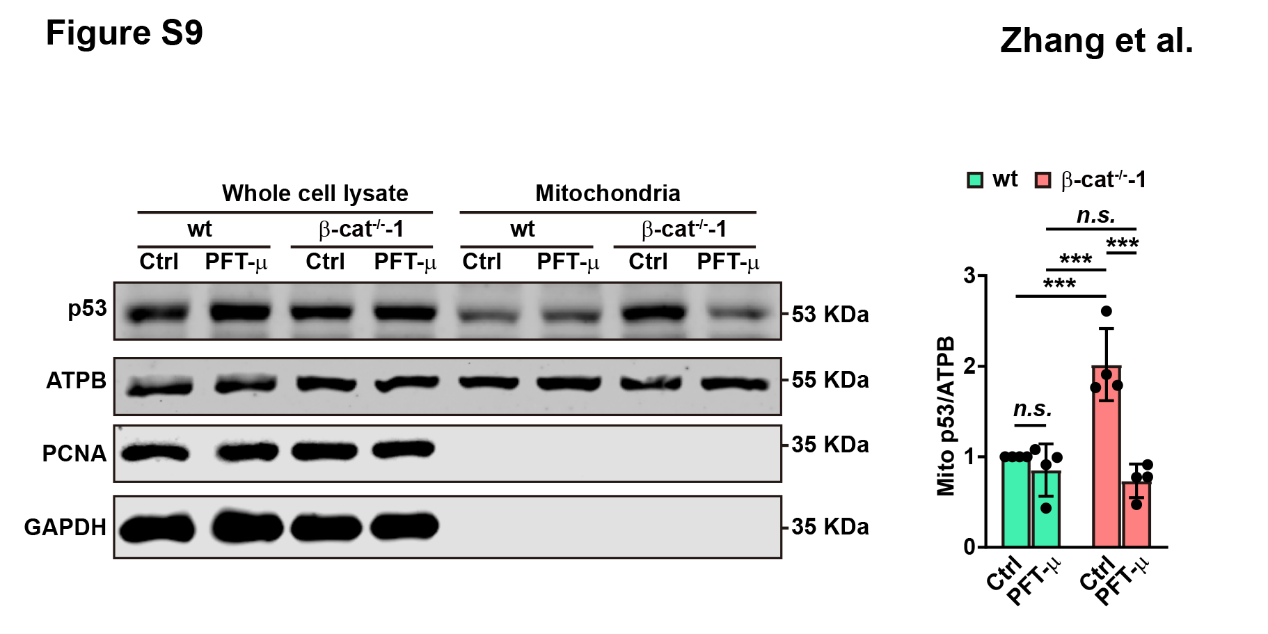


**Figure S9. The** **mitochondrial translocation of p53 in cells treated with PFT-μ during PS induction.**

Left panel, the western blot analysis of mitochondrial translocation of p53. Right panel, the summarized data. Cells were harvested at 2 hours after PS induction. PFT-μ was added at the initiation of PS induction.


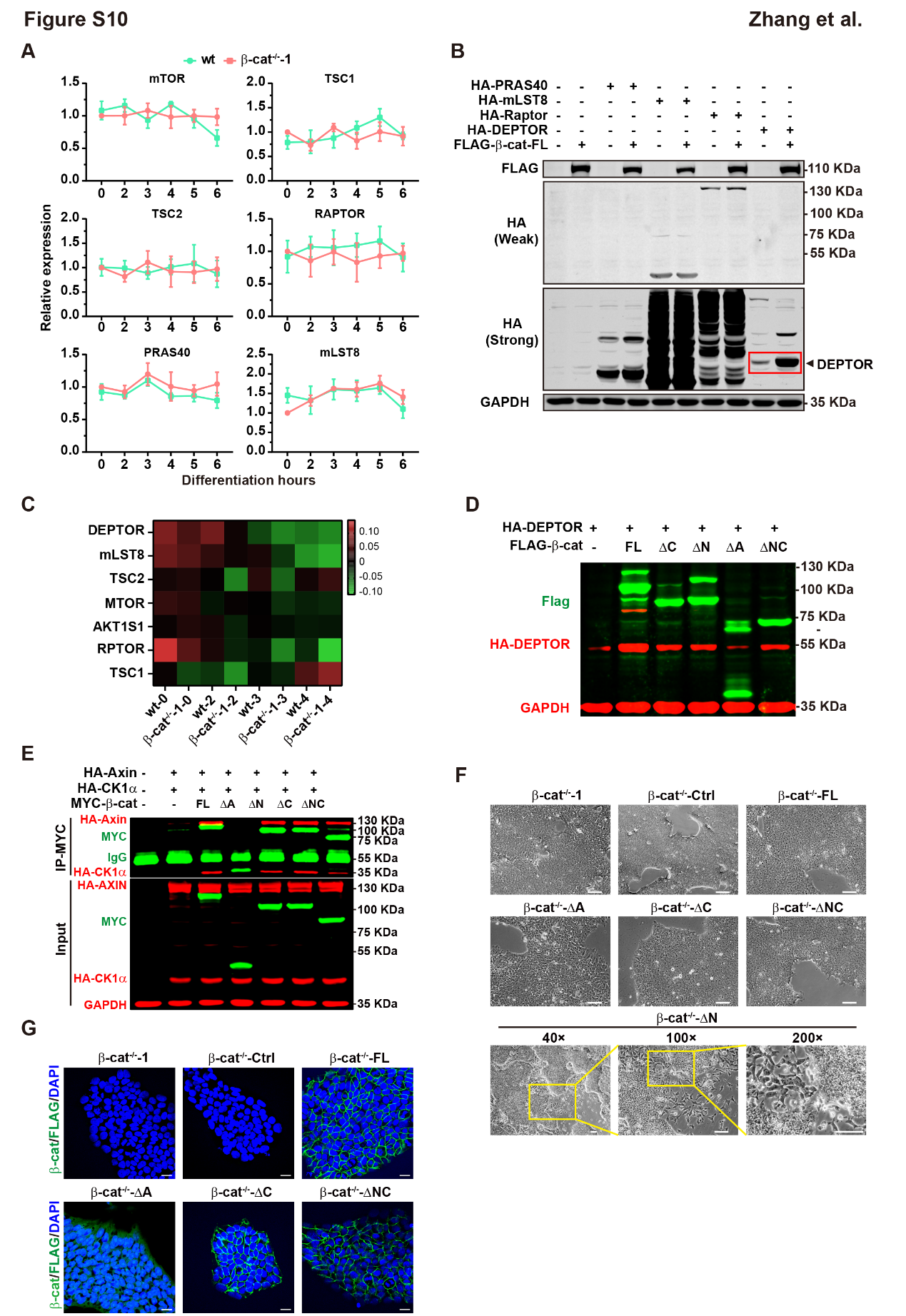


**Figure S10. The armadillo repeat domain of β-catenin is responsible for stabilizing of DEPTOR, related to Figure 6.**

**(A)** Summarized western blot data of mTOR, TSC1, TSC2, Raptor, RPAS40, and mLST8 proteins during PS induction. Data were normalized to GAPDH. *n* = 3.

**(B**) Western blot analysis of protein levels of PRAS40, mLST8, Raptor, and DEPTOR with or without β-catenin overexpression. Short exp., exposure with short time; long exp., exposure with long time. β-catenin is fused with FLAG tag, and PRAS40, mLST8, Raptor, and DEPTOR are fused with HA tag. GAPDH is used as loading control.

**(C**) Heatmap of the mRNA level of 7 mTOR modulator/component genes.

**(D**) Western blot analysis of the protein level of DEPTOR in HEK293T cells transfected with plasmids containing indicated forms of β-catenin. β-catenin and its truncations were fused with FLAG tag, and DEPTOR were fused with HA tag. GAPDH is used as loading control.

**(E**) Interaction analysis of CK1α and different versions of β-catenin in hESCs.

**(F**) Morphology of β-cat^-/-^-1 hESCs overexpressed with β-cat-FL, β-cat-ΔA, β-cat-ΔC, β-cat-ΔNC, and β-cat-ΔN. Scale bar, 100 μm. 40×, 100× and 200×, different magnification.

**(G**) Immunofluorescence staining of β-cat^-/-^-1 hESCs with β-cat-FL, β-cat-ΔA, β-cat-ΔC, β-cat-ΔNC, β-cat-ΔN overexpression. The ΔA, ΔC, and ΔNC forms of β-catenin were detected by the FLAG antibody as mentioned in the METHOD- Immunofluorescence staining. Scale bar, 20 μm.

**Supplementary Tables**

**Table S1. Analyses of enriched biological processes of GO term and KEGG pathways for the DEGs between the wt and β-cat^-/-^-1 cells at 3 and 4 hours after PS induction**

See “CDDISCOVERY-24-2507-TR1-Table S1.xlsx” file.

**Table S2. The Top 100 enriched biological processes related to Figure 3E**

See “CDDISCOVERY-24-2507-TR1-Table S2.xlsx” file.

**Table S3. The LC-MS analysis for co-precipitated proteins with β-catenin**

See “CDDISCOVERY-24-2507TR1-Table S3.xlsx” file.

**Table S4. gRNAs and primers for mutation detection (related to Figure S2 and S4)**

| **Name** | **Sequences** |
| --- | --- |
| gRNA-*CTNNB1* | 5’-AAAAGCGGCTGTTAGTCACTGG-3’, PAM |
| gRNA-*TP53* | 5’-CATTGTTCAATATCGTCCGGGG-3’, PAM |
| Seq-*CTNNB1*-F | 5’-AACAAGCCACCAGCAGGAAT-3’ |
| Seq-*CTNNB1*-R | 5’-TAGCAGCTCGTACCCTCTGA-3’ |
| Seq-*TP53*-F | 5’-CCCAGGGTTGGAAGTGTCTC-3’ |
| Seq-*TP53*-R | 5’-GCTGCCCTGGTAGGTTTTCT-3’ |

**Table S5. Primers for qRT-PCR (related to Figure 2, Figure S1 and Figure S5)**

| **Gene** | **Forward primer (5’ to 3’)** | **Reverse primer (5’ to 3’)** |
| --- | --- | --- |
| *POU5F1* | AGTGAGAGGCAACCTGGAGA | ACACTCGGACCACATCCTTC |
| *EOMES* | CAACATAAACGGACTCAATCCCA | ACCACCTCTACGAACACATTGT |
| *MIXL1* | CCGAGTCCAGGATCCAGGTA | CTCTGACGCCGAGACTTGG |
| *TBXT* | CAGTGGCAGTCTCAGGTTAAGAAGGA | CGCTACTGCAGGTGTGAGCAA |
| *MESP1* | GCTCTGTTGGAGACCTGGAT | CAGTCTGCCAAGGAACCACT |
| *ISL1* | ATCAGGTTGTACGGGATCAAATG | ATGTGATACACCTTGGAGCG |
| *NKX2-5* | CAAGTGTGCGTCTGCCTTT | CAGCTCTTTCTTTTCGGCTCTA |
| *TNNT2* | ACAGAGCGGAAAAGTGGGAAG | TCGTTGATCCTGTTTCGGAGA |
| *FAS* | AGATTGTGTGATGAAGGACATGG | TGTTGCTGGTGAGTGTGCATT |
| *PUMA* | GACCTCAACGCACAGTACGAG | AGGAGTCCCATGATGAGATTGT |
| *CDKN1A* | CGATGGAACTTCGACTTTGTCA | GCACAAGGGTACAAGACAGTG |
| *AXIN2* | TACACTCCTTATTGGGCGATCA | TTGGCTACTCGTAAAGTTTTGGT |
| *GAPDH* | GGAGCGAGATCCCTCCAAAAT | GGCTGTTGTCATACTTCTCATGG |

**Table S6. Key reagents used in this study.**

| **REAGENT or RESOURCE** | **SOURCE** | **IDENTIFIER** |
| --- | --- | --- |
| **Antibodies** | | |
| Anti-β-Catenin | BD Biosciences | Cat: 610154 |
| Anti-p53 (DO-1) | Santa Cruz | Cat: SC-126 |
| Anti-BAX | Proteintech | Cat: 50599-2-Ig |
| Anti-E-cadherin | BD Biosciences | Cat: 610404 |
| Anti-phospho-P53(Ser15) | Cell signaling | Cat: 9284S |
| Anti- Acetyl-p53 (Lys382) | Cell signaling | Cat: 2525S |
| Anti-β-Actin | Boster | Cat: BM0627 |
| Anti-p-s6k (phospho T389) | Abcam | Cat: ab2571 |
| Anti-P70 S6 Kinase | Cell signaling | Cat: 2708S |
| Anti- Phospho-4E-BP1 (Thr37/46) | Cell signaling | Cat: 2855S |
| Anti-eIF4EBP1 | Abcam | Cat: ab2606 |
| Anti-GAPDH | Proteintech | Cat: 10494-1-AP |
| Anti-LC3 | Sigma-Aldrich | Cat: L8918 |
| Anti-ATPB | Abcam | Cat: ab14730 |
| Anti-PCNA | Santa Cruz | Cat: sc-56 |
| Anti-mTOR | Cell signaling | Cat: 2983S |
| Anti-TSC1 | Cell signaling | Cat: 4906 |
| Anti-TSC2 | Cell signaling | Cat: 4308 |
| Anti-RAPTOR | Cell signaling | Cat: 2280S |
| Anti-PRAS40 | Abcam | Cat: ab133584 |
| Anti- GβL(MLST8) | Cell signaling | Cat: 3274S |
| Anti-DEPTOR | Cell signaling | Cat: 11816S |
| Anti-FLAG(M2) | Sigma-Aldrich | Cat: F-1804 |
| Anti-HA | Cell signaling | Cat: 3724S |
| Anti-Myc | Santa Cruz | Cat: SC-40 |
| Anti-CK1α | Cell signaling | Cat: 2655S |
| Anti-α-Actinin | Abcam | Cat: ab9465 |
| Anti-CTNT | Abcam | Cat: ab8295 |
| Anti-OCT4 | Abcam | Cat: ab19857 |
| Anti-SOX2 | R&D Systems | Cat: AF2018 |
| Anti-Mouse IgG (H+L) Alexa Fluor Plus 488 | Invitrogen | Cat: A32723 |
| Anti-Rabbit IgG (H+L) Alexa Fluor 555 | Invitrogen | Cat: A21428 |
| IRDye 680LT Donkey anti-Rabbit IgG (H + L) | LI-COR BIOSCIENCES | Cat: 926-68023 |
| IRDye 800CW Donkey anti-Mouse IgG (H + L) | LI-COR BIOSCIENCES | Cat: 926-32212 |
| **Chemicals, peptides, and recombinant proteins** | | |
| CHIR99021 | Stemcell Technologies | Cat: 72054 |
| IWR-1 | Merck | Cat: 681669-10MG |
| RPMI1640 | Gibco | Cat: C11875500CP |
| mTeSR™1 | Stemcell Technologies | Cat: 05850 |
| Matrigel | Corning | Cat: 354277 |
| Bovine Serum Albumin | Sigma-Aldrich | Cat: A1933-100G |
| AnnexinV-FITC | BD Biosciences | Cat: 556419 |
| Propidium iodide solution | Sigma-Aldrich | Cat: P4864 |
| L-Ascorbic acid 2-phosphate sesquimagnesium salt hydrate | Sigma-Aldrich | Cat: A8960 |
| DAPI | ThermoFisher | Cat: 62247 |
| Nutlin-3a | Selleck | Cat: S8059 |
| Pifithrin-α | Selleck | Cat: S2929 |
| Pifithrin-μ | Selleck | Cat: S2930 |
| Rapamycin | Selleck | Cat: S1039 |
| RAD001 | Selleck | Cat: S1120 |
| MG132 | Selleck | Cat: S2619 |
| MitoTracker™ Green FM | Invitrogen | Cat: M7514 |
| LysoTracker™ Red DND-99 | Invitrogen | Cat: L7528 |
| FCCP | Sigma-Aldrich | Cat: C2920 |
| Y-27632 | Stemcell Technologies | Cat: 72308 |
| BMP4 | R&D Systems | Cat: 314-BP/CF |
| Activin A | R&D Systems | Cat: 338-AC-050 |

**Supplementary videos**

**Video S1. Beating cardiomyocytes from H1 hESCs at differentiation day 10, related to Figure S1.**

**Video S2. Beating cardiomyocytes from β-cat^-/-^-1 hESCs overexpressing full-length β-catenin at differentiation day 15, related to Figure 7.**
