# Supplementary material for: β-catenin safeguards cell survival via a transcription-independent mechanism during the induction of primitive streak from hESCs
Source: Cell Death Discov. 2025 Jul 2;11:300. doi: 10.1038/s41420-025-02559-w (PMC12222672; doi:10.1038/s41420-025-02559-w)

## Figure 1

## Figure 1B

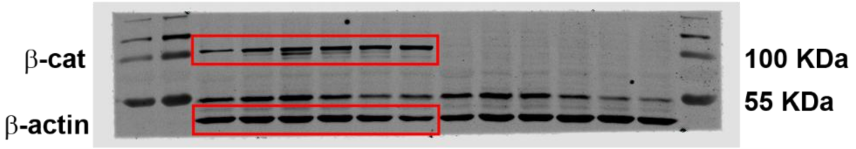


## Figure 2

## Figure 2D

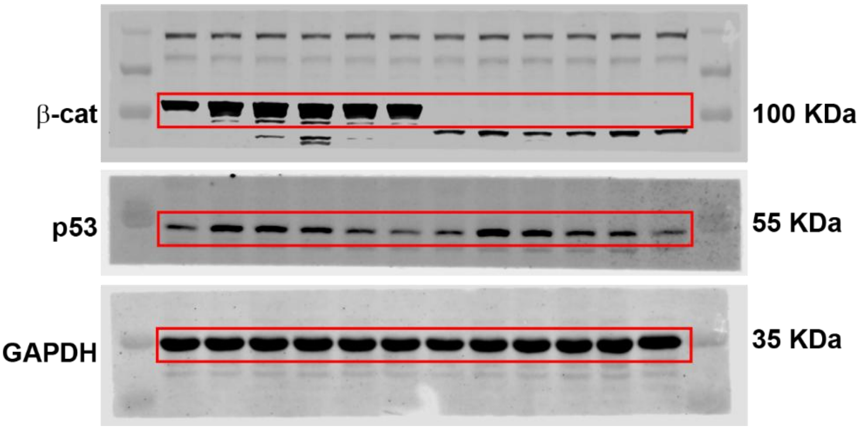

## Figure 4

### Figure 4A

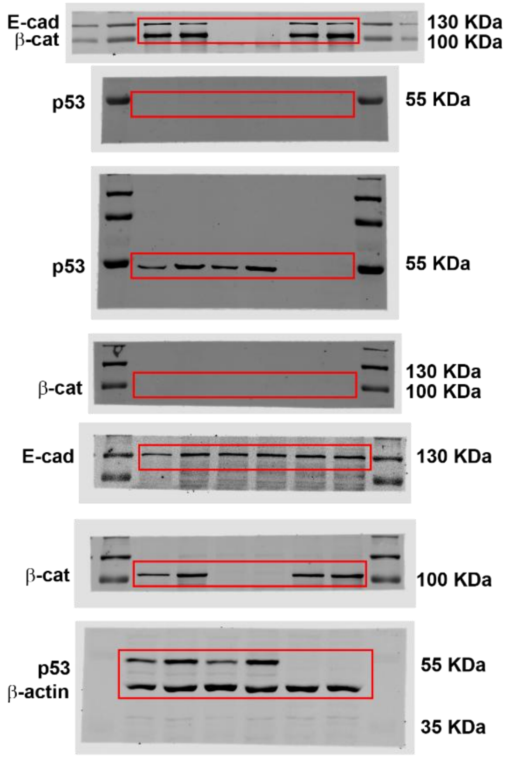


### Figure 4B

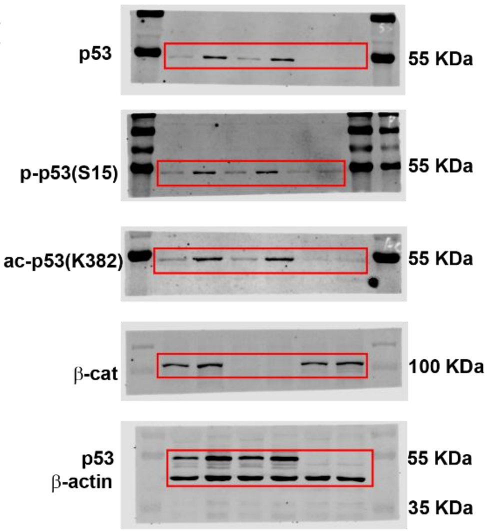


### Figure 4D

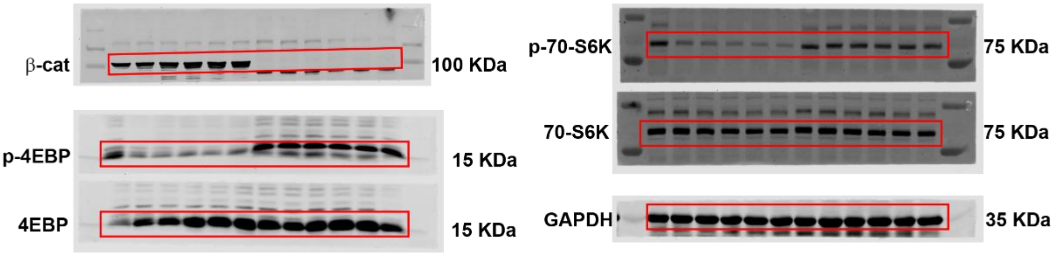

# Figure 5

## Figure 5A

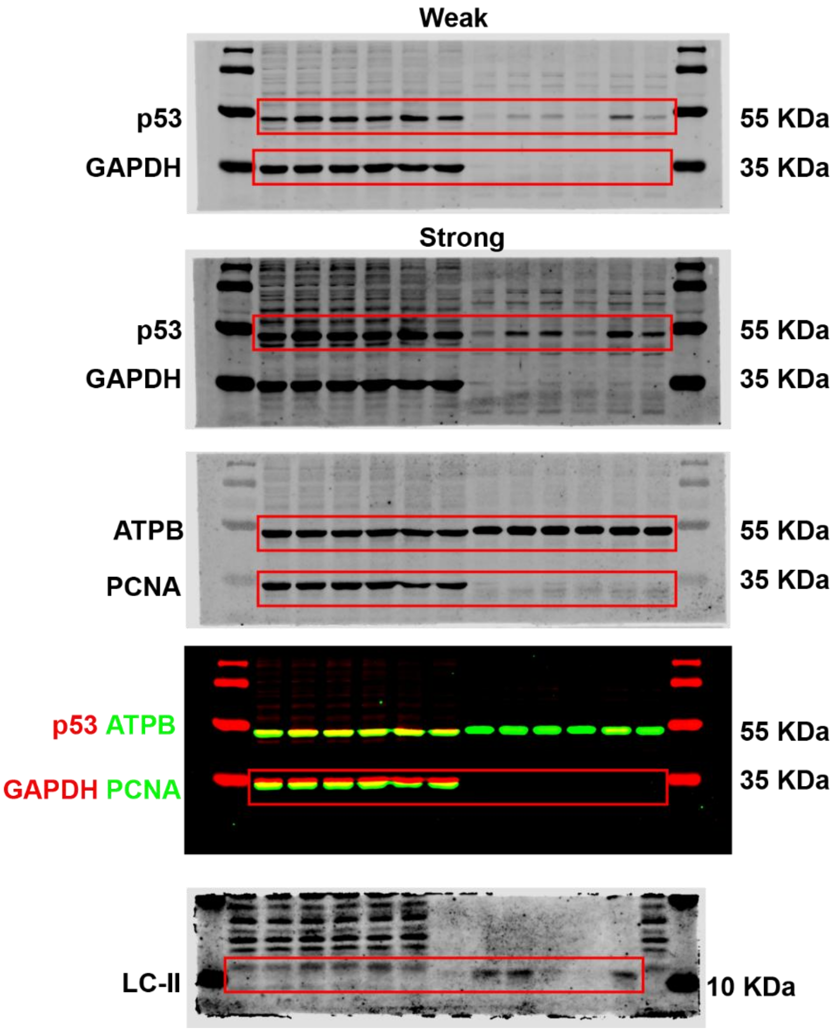

## Figure 6

### Figure 6A

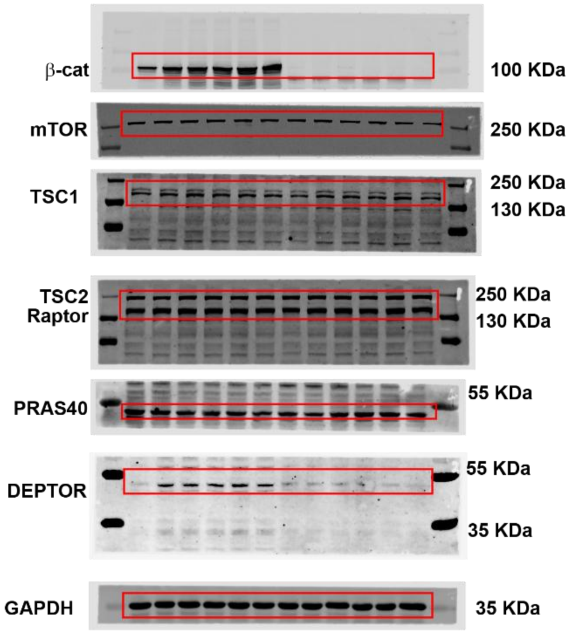


### Figure 6C

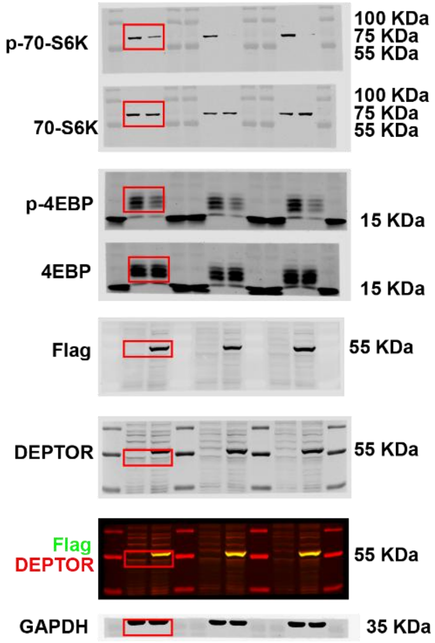


### Figure 6E

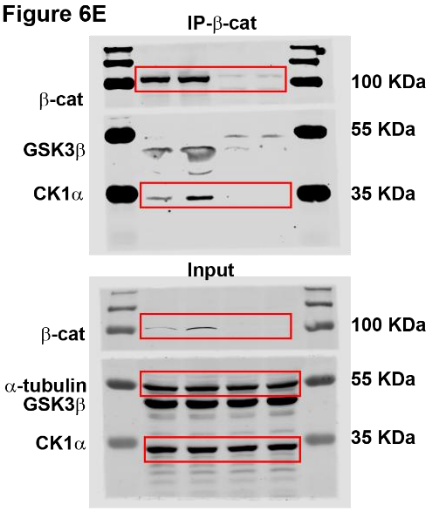


### Figure 6F

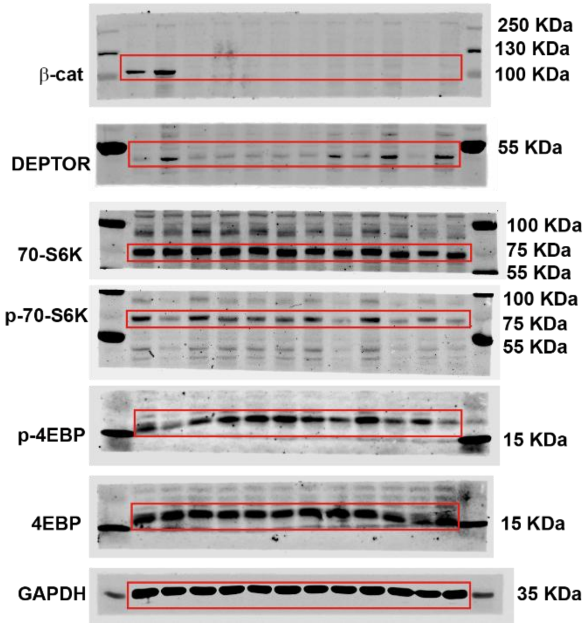

## Figure 6

### Figure 6G

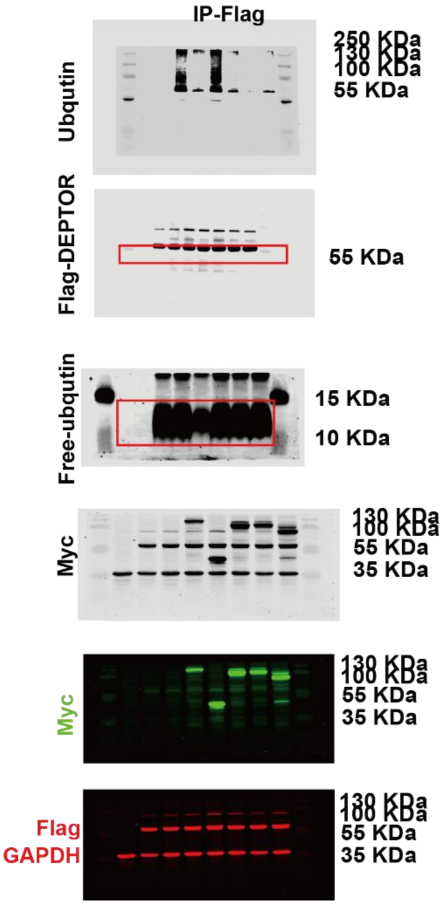


### Figure 6H

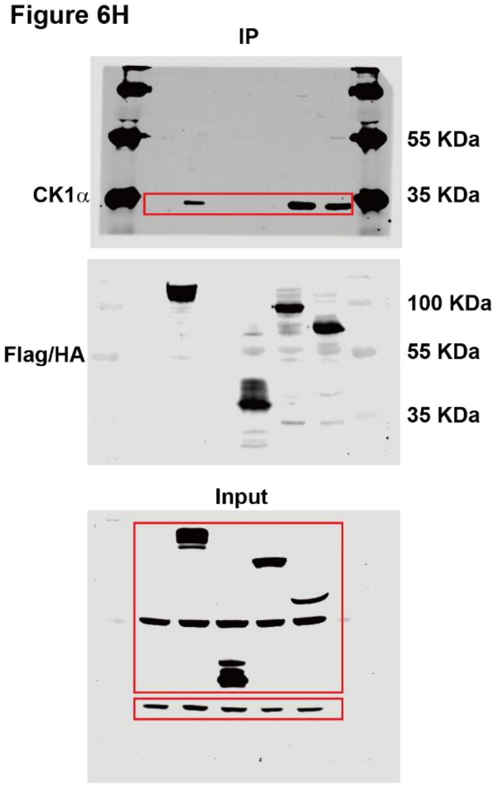

## Figure 6

## Figure 6I

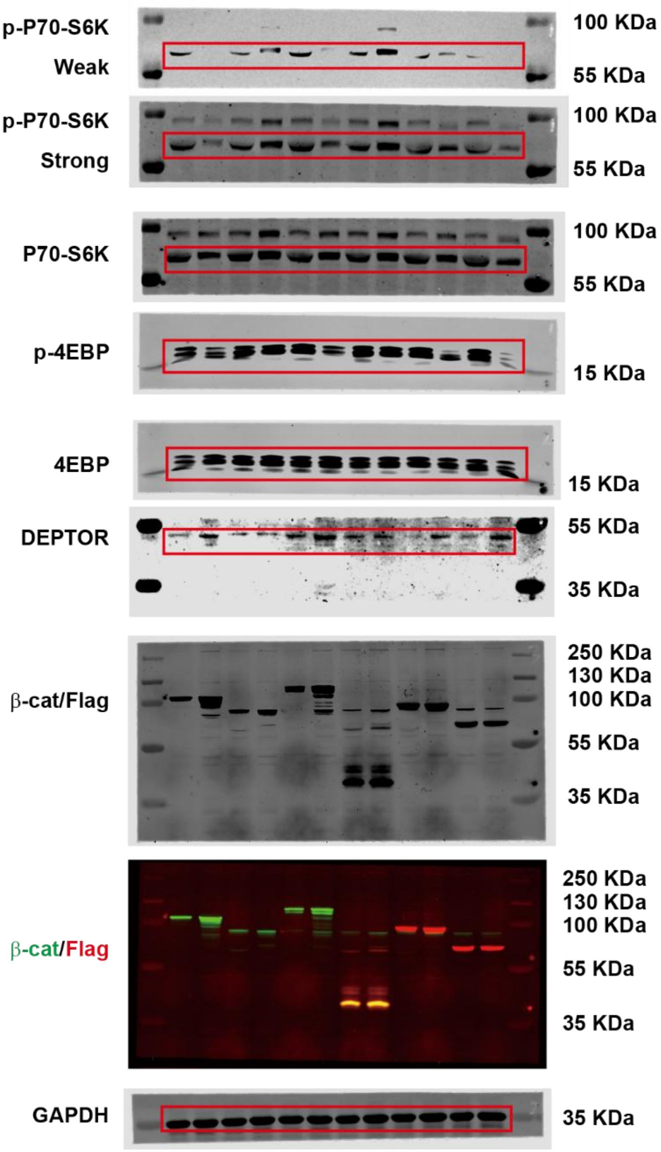

## Figure S2B

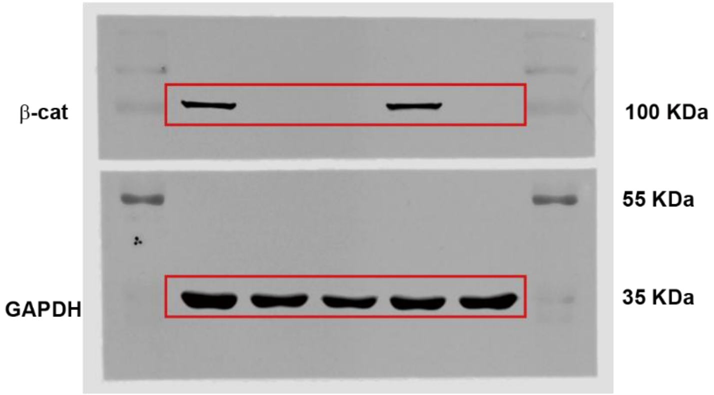


## Figure S4E

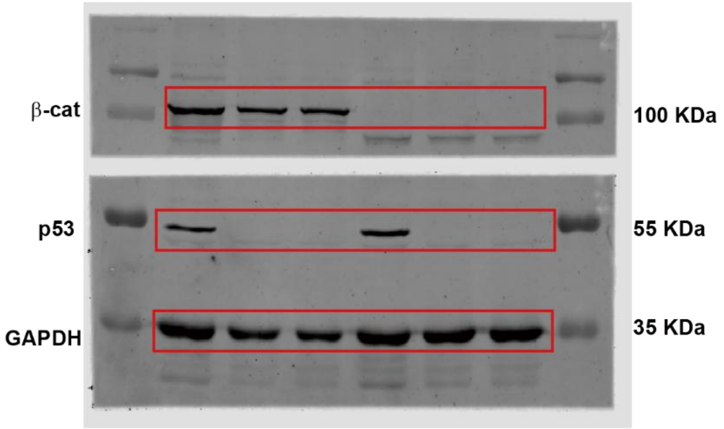


## Figure S7D

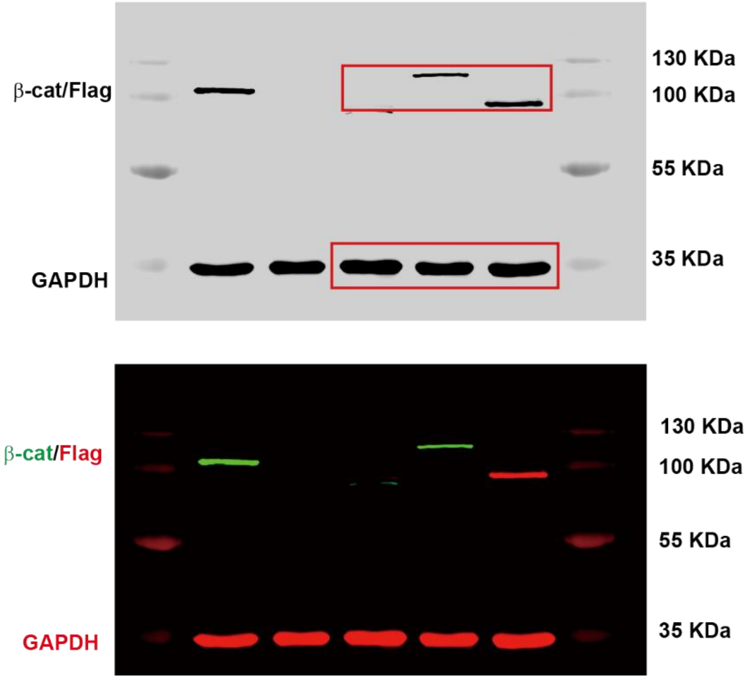

## Figure S9

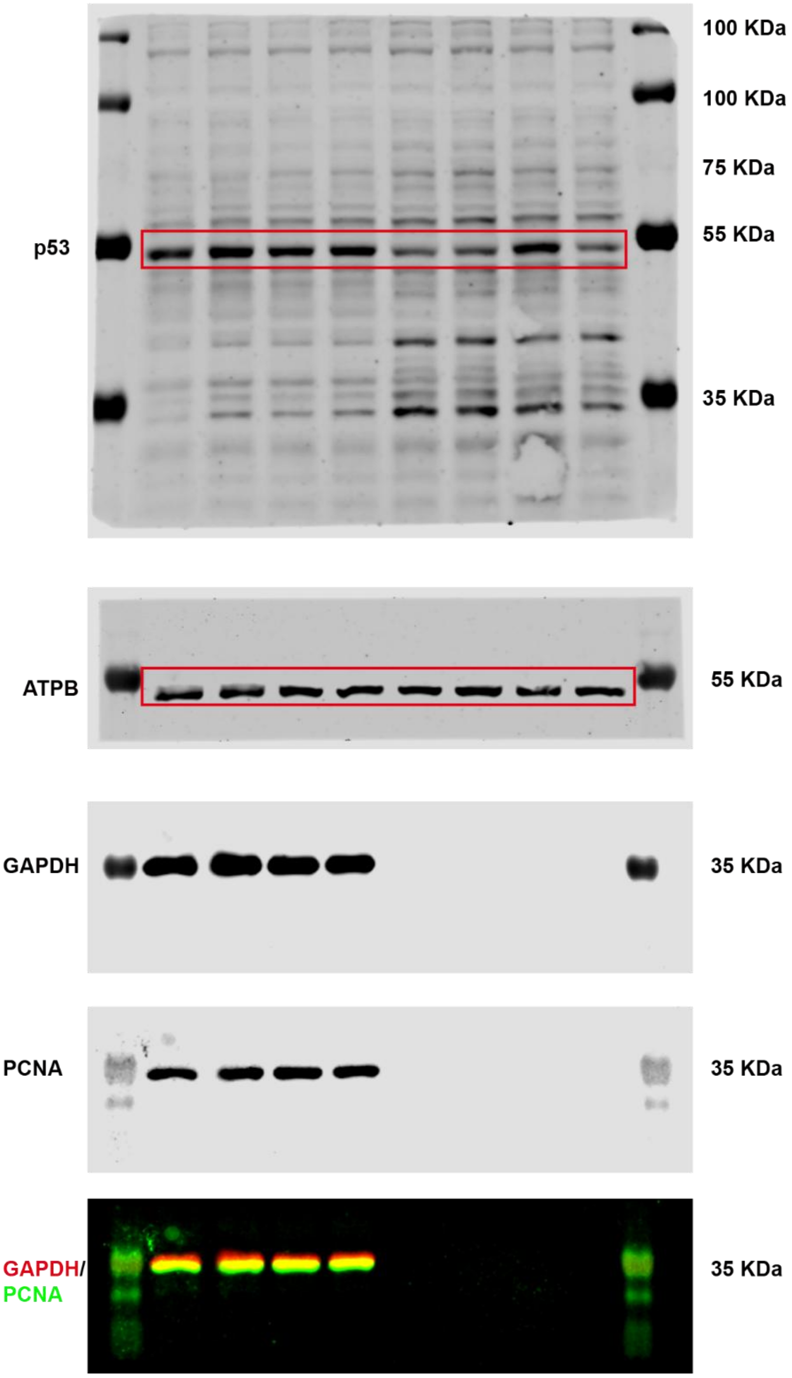

p53
100 KDa
100 KDa
75 KDa
55 KDa
35 KDa
ATPB
55 KDa
GAPDH
35 KDa
PCNA
35 KDa
GAPDH/
PCNA
35 KDa

## Figure S10B

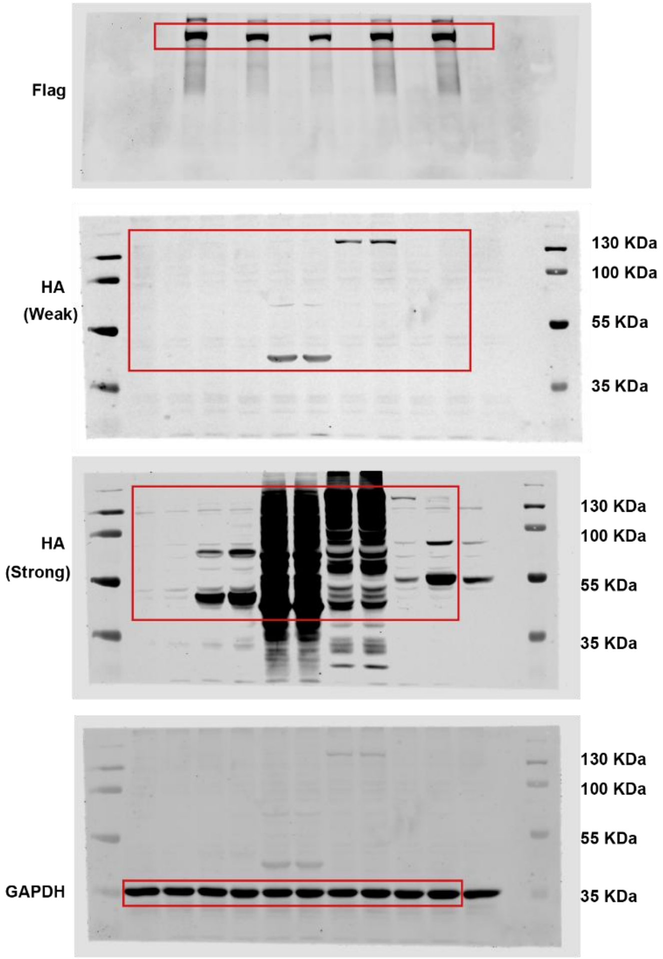


## Figure S10E

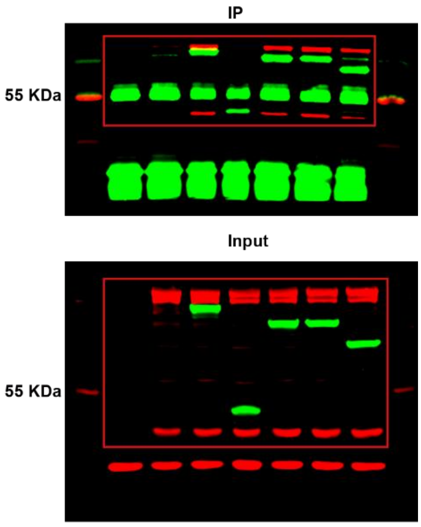


## Figure S10D

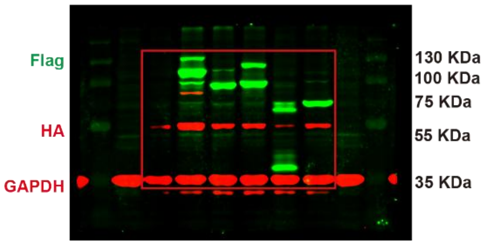

Supplement: Supplementary file 7 — Original western blot images [file 41420_2025_2559_MOESM7_ESM.pdf]
